# Legal and regulatory instruments for NCD prevention: a scoping review and descriptive analysis of evaluations in OECD countries

**DOI:** 10.1186/s12889-024-18053-4

**Published:** 2024-02-29

**Authors:** Maddie Heenan, Ashleigh Chanel Hart, Katherine Cullerton, Stephen Jan, Janani Shanthosh

**Affiliations:** 1 The George Institute for Global Health, University of New South Wales, Level 5/ 1 King St Newtown, Sydney, NSW 2042 Australia; 2 The Australian Prevention Partnership Centre, Level 3, 30C Wentworth Street, Glebe, NSW 2037 Australia; 3 Australian Human Rights Institute, University of New South Wales, Sydney, NSW 2052 Australia; 4 School of Public Health, University of Queensland, 266 Herston Rd, Herston, QLD 4006 Australia

**Keywords:** NCD prevention, Public health law, Regulation, Evaluation, Tobacco, Food, Alcohol, Environmental pollutants

## Abstract

**Context:**

Public health law is an important tool in non-communicable disease (NCD) prevention. There are different approaches available for achieving policy objectives, including government, co-, quasi- and self-regulation. However, it is often unclear what legal design features drive successes or failures in particular contexts. This scoping review undertakes a descriptive analysis, exploring the design characteristics of legal instruments that have been used for NCD prevention and implemented and evaluated in OECD countries.

**Methods:**

A scoping review was conducted across four health and legal databases (Scopus, EMBASE, MEDLINE, HeinOnline), identifying study characteristics, legal characteristics and regulatory approaches, and reported outcomes. Included studies focused on regulation of tobacco, alcohol, unhealthy foods and beverages, and environmental pollutants.

**Findings:**

We identified 111 relevant studies evaluating 126 legal instruments. Evaluation measures most commonly assessed implementation, compliance and changes to the built and lived environment. Few studies evaluated health or economic outcomes. When examining the design and governance mechanisms of the included legal instruments, government regulation was most commonly evaluated (*n* = 90) and most likely to be reported effective (64%). Self-regulation (*n* = 27) and quasi-regulation (*n* = 5) were almost always reported to be ineffective (93% and 100% respectively). There were few co-regulated instruments evaluated (*n* = 4) with mixed effectiveness. When examining public health risks, food and beverages including alcohol were more likely to be self- or quasi-regulated and reported as ineffective more often. In comparison, tobacco and environmental pollutants were more likely to have government mandated regulation. Many evaluations lacked critical information on regulatory design. Monitoring and enforcement of regulations was inconsistently reported, making it difficult to draw linkages to outcomes and reported effectiveness.

**Conclusions:**

Food and alcohol regulation has tended to be less successful in part due to the strong reliance on self- and quasi-regulation. More work should be done in understanding how government regulation can be extended to these areas. Public health law evaluations are important for supporting government decision-making but must provide more detail of the design and implementation features of the instruments being evaluated – critical information for policy-makers.

**Supplementary Information:**

The online version contains supplementary material available at 10.1186/s12889-024-18053-4.

## Policy points

Government regulation is reported as more effective than co-regulation, quasi-regulation or self-regulation. Voluntary approaches, including voluntary government regulation, are reported less effective due to low uptake and limited accountability. In public health law mandated government regulation should be strived for.

Many public health law evaluations are lacking critical design information for policy makers. This may make it difficult to learn from successes or failures and replicate interventions in other jurisdictions.

## Introduction

Non-communicable disease (NCD) prevention is complex and effective prevention requires action across multiple risk factors and determinants, and multiple policy areas. Law is an established instrument of health promotion and health protection, particularly in the area of NCD-prevention [1, 2]. Public health law, encompasses traditional legal functions and policy processes, providing governments with the authority and scope of power to improve population health [3]. Many international successes in NCD prevention are grounded in public health law, including the regulation of tobacco, alcohol, unhealthy foods and beverages, and environmental pollutants.

Governments have different legal and regulatory approaches at their disposal, using legislation, regulation and policies to achieve particular health outcomes. Policy can be defined as the plan of action, and law and regulation as the different implementation tools to achieve the policy objective [4]. Different regulatory approaches can be employed by governments to guide or coerce industry behaviour for public health benefits [5]. These different approaches or forms of regulation can be used in response to different contexts (see definitions in Table 1) [6]. For example, there are costs associated with implementing and enforcing regulations, as such the government mandate is often to look for a non-regulatory solution first to help reduce these costs, allowing industries to self-regulate [7]. Governments may step in and regulate when self-regulation is proving to be ineffective.

**Table 1:** Examples of different forms of public health regulations (adapted from Reeve 2013) [6]

Form of regulation	Characteristics	Example
**Self-regulation**	Industry formulates rules and codes of conduct, industry bodies solely responsible for enforcement.	Examples include many advertising codes such as the Beer Institute Advertising and Marketing Code or The EU Pledge for Responsible Food Marketing.
**Quasi-regulation**	Government influences the development and implementation of self-regulation without using legislation; e.g., government threatens industry with legislation or points to existence of self-regulation as justification for not regulating.	Examples include food reformulation schemes such as Australia’s Food and Health Dialogue.
**Co-regulation**	Industry and government jointly develop, administer, and enforce a regulatory regime; e.g., governments prescribe self-regulation but allow industry to develop terms, delegate power to industry to regulate and enforce codes.	Examples include liquor licensing arrangements such as New Zealand’s Alcohol Licensing Trusts.
**Explicit government regulation**	Government develops, administers and enforces the regulation; e.g. primary and subordinate legislation.	Examples include the Philadelphia Lead Court and Health Code, United Kingdom (UK) Chemical (Hazard Information and Packaging for Supply) Regulations 2009 and the Swizerland Tobacco Control Act.

Responsive regulation supports this incremental approach typically starting with voluntary self-regulation and moving towards explicit government regulation if policy objectives are not achieved and/or industry fails to cooperate [8]. This approach can move up and down the scale, responding to industry characteristics and current contexts, including political, social and economic, and correcting any deficiencies [9]. At the same time, governments must remain accountable for the public’s health and consider the conflicts of interest in certain industries’ self-regulating.

This is consistent with the Organisation for Economic Co-operation and Development (OECD) seven principles of best practice regulation: (1) role clarity; (2) preventing undue influence and maintaining trust; (3) decision-making and governing body structure for independence; (4) accountability and transparency; (5) engagement; (6) funding; (7) performance evaluation [10]. 

Effective regulation can improve population health outcomes and lead to economic and social benefits. The way that laws are designed and implemented, and the structures that surround them, can impact our health in positive and negative ways [11]. Law is a determinant of health that can impact all the social determinants; when well-designed they can improve health systems and the built and natural environments in which we live, work and play, or they can entrench disadvantage, stigma and discrimination when poorly designed, implemented or enforced [11]. It is therefore important to understand the circumstances and design features of effective public health law in order to replicate them and ensure improved health outcomes rather than further entrenching disadvantage.

Theoretically a lot is known about how and why public health law works. But empirical public health law research is fragmented and lacking a culture of evaluation. Public health law research is diverse focusing on policymaking, mapping, implementation, intervention outcomes and mechanism outcomes [12]. However, the concept of public health law research is newly defined and a lot of research on public health law does not consider itself as ‘public health law research’, but as either scientific research or legal scholarship– two distinct fields of research [3]. As such their evaluations, approaches and information included in publications are different: scientific research centres outcomes and impact but lacks a focus on the regulatory design features that facilitate or create those outcomes, while legal scholarship or regulatory theory centres design features but lacks a specific focus on outcomes and impact. This means that policymakers, prevention researchers and public health lawyers have limited opportunity to learn from the design features of public health law that drive successes or failures in particular contexts and under specific governance and financing arrangements. Additionally, research on major public health risk factors are often conducted in silos, focusing on a single risk factor. Little work has been undertaken that synthesises the evidence across different risk factors to generate lessons for decision makers. While public health researchers and advocates often refer to lessons learnt in tobacco control, it is unclear whether the same regulatory approaches are used across different public health risks and what makes them effective.

This scoping review attempts to address this gap by describing the existing evidence and exploring the characteristics of legal and regulatory instruments for NCD prevention that have been implemented and evaluated in OECD countries. The OECD is commonly used as a comparator in policy research with member countries typically having similar income levels and social, economic, political and legal structures [10]. For the purposes of this scoping review, we focus on OECD countries to provide a reasonable comparison when looking at the design and implementation of public health law. Looking at the common public health risks of tobacco [13], alcohol [14], environmental pollutants [15] and unhealthy foods and beverages [16], we aim to map the literature of public health law, and look at how instruments are designed, implemented and whether they are reported to be impactful and effective within the OECD.

In achieving the above aim we wanted to answer the following:

- What legal and regulatory instruments are used to regulate tobacco, alcohol, unhealthy foods and beverages, and environmental pollutants for NCD prevention, how are they designed and implemented, and are they reported as effective?
- Sub-questions:
- What type of evaluations are undertaken?
- What type of instruments are being evaluated for different risk factors, and where?
- How are they regulated, governed, monitored and enforced?
- What is the reported effectiveness, if any, of different regulatory designs?

## Methods

A scoping review was undertaken of relevant peer-reviewed and grey literature, guided by the Arksey and O’Malley Framework [17], to identify regulatory reviews and empirical evaluations of legal and regulatory instruments used in public health. Scoping reviews are used to identify key concepts or factors related to a topic and address broader review questions than traditionally more specific systematic reviews [18]. They are also a useful method of evidence synthesis when the literature is complex and heterogenous, such as in this instance regarding public health policy and regulatory theory. Additionally, this type of evidence synthesis is particularly useful for decision-makers, providing an overview of research in a given policy area or in regards to types of interventions researched [19]. 

### Search

MH conducted an initial search of databases in collaboration with medical and legal librarians to identify index terms and test the search strategy (see Additional file 1). Four databases were searched, two biomedical, one social sciences/ health sciences and one legal database: MEDLINE, EMBASE, Scopus and HeinOnline. The same concepts and key terms were search across all databases. Inclusion and exclusion criteria are provided in Table 2.

**Table 2:** Inclusion and exclusion criteria

	Inclusion	Exclusion
**Publication date range**	• January 2000– July 2020	• < 2000 or > July 2020
**Language**	• English	• Non-English
**Countries**	• OECD countries • Cross-country studies involving the above countries	• Non-OECD counties
**Type of publication**	• Original studies or empirical research	• Letters • Editorials • Commentaries • Notes • Books and book reviews
**Type of research**	• Quantitative • Qualitative • Mixed methods	
**Study design**	• Evaluations, regulatory reviews, legal reviews that report effectiveness of a legal or regulatory instrument	• Systematic reviews • Meta-analyses • Policy audits • Evaluations, regulatory reviews, legal reviews that do not report effectiveness
**Risk factor**	• Tobacco • Unhealthy foods and beverages (high in salt, sugar or fat) • Alcohol • Environmental pollutants (e.g. lead, chemicals, air pollution)	• Pharmaceuticals, patents, medical devices & therapies, nano-technologies, dietary supplements • Infectious diseases, injury, gun safety, road safety, food safety, mental health, illicit drugs • animal welfare, livestock care, agricultural practice that doesn’t relate to included risks
**Legal or regulatory instrument**	• Adopted national, state, local or regional laws and regulations. • Information about the ‘instrument’ being evaluated (minimum information required: type, aim, who has ownership i.e. gov/industry, jurisdiction, target population).	• International laws or frameworks, trade related laws and agreements, laws regarding medical practice and clinical guidelines. • Proposed laws or regulations that have not been adopted. • Evaluations of regulation that do not provide enough detail on the instrument (i.e. objectives, design and implementation features).

### Screening

MH screened each of the retrieved titles and abstracts against the eligibility criteria using Covidence. A research assistant double screened 15% to validate the screening strategy. There was a high level of agreement and any identified discrepancies were discussed and resolved. Full text screening was undertaken by MH and ACH, reasons for exclusion were recorded and discrepancies discussed and resolved between authors. All included studies were double screened by MH.

### Data extraction and analysis

MH and JS developed and tested the data extraction tool (see Additional file 2). Included studies were then extracted by MH and ACH. We were interested in the general characteristics of the research, including methods and evaluation type; characteristics of the legal instrument, including regulatory form; evaluation outcomes varying from health impacts, compliance rates and whether laws were implemented as intended; and reported effectiveness.

We determined the type of evaluation adapting the Bauman & Nutbeam evaluation definitions for health promotion programs because public health law is a type of health promotion initiative using legal and regulatory approaches [20]. When referring to process evaluations we take a broader approach because of the broad nature of public health law evaluations and reviews. While we acknowledge that process evaluations have a specific methodology, in this paper we are trying to bring together diverse literature and as such use a more encompassing definition of evaluations on process. This is because legal and regulatory reviews are often excluded because they do not fit classical empirical evaluation definitions.

The reported effectiveness of the evaluated instrument was recorded as effective, partially effective or not effective as per the reported findings of the authors. It was not part of this study’s objective to critique the evaluation design nor the effectiveness of the legal instruments in the included studies, but to record the type of study design used and effectiveness of the instrument as reported by the evaluation team. This scoping review describes and synthesises the types of legal and regulatory evaluations undertaken and their reported outcomes, by nature it is not intended to be a rigorous quantitative synthesis of the rigor of legal evaluations. An additional file details a data dictionary for terms used in data extraction and analysis (see Additional file 3).

## Results

### Search and selection of studies

**Fig. 1:**
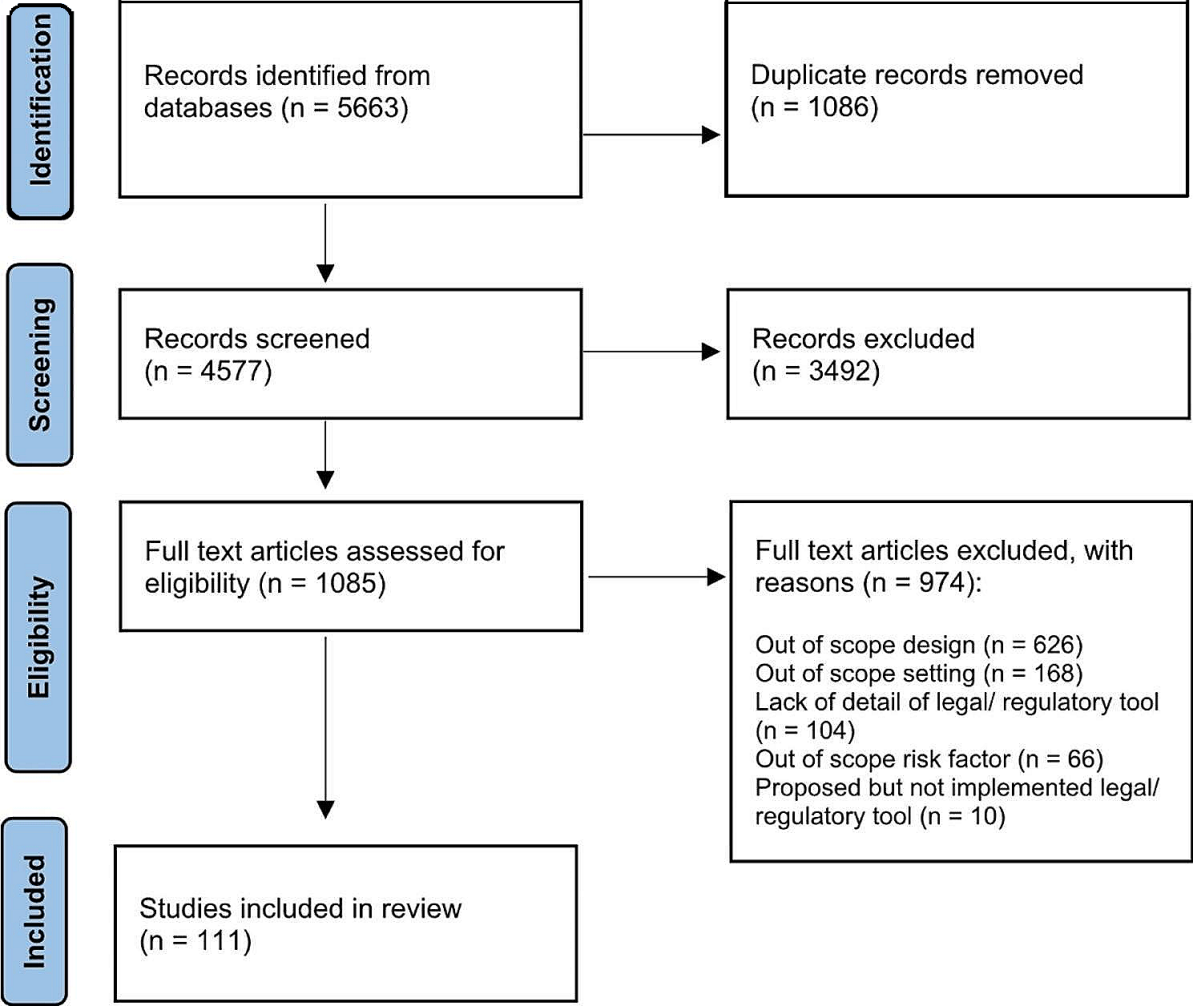
PRISMA diagram

A total of 5663 articles were identified. Duplicates (*n* = 1086) were removed leaving 4577 titles and abstracts to be screened for relevance with a further 3492 excluded and the remaining 1085 studies assessed for full-text eligibility (Fig. 1). Many articles identified via HeinOnline were legal reviews that lacked an abstract and were found to study a public health risk and/or setting outside the scope of this research. The majority of studies were excluded because they had a study design outside the scope of this research. This may be because they were describing a policy problem, simply providing commentary, or focused on epidemiology without analysing or evaluating a legal or regulatory instrument. Many legal analyses provided comprehensive descriptions of laws but did not review or evaluate outcomes and therefore were also excluded. This is compared to public health articles that evaluated outcomes but did not provide sufficient detail of the instrument’s design or implementation. As the aim was to explore the characteristics of legal and regulatory instruments, including governance and enforcement mechanisms, and what makes them effective, it was essential for included articles to provide details of the instrument’s design and implementation. Additionally, some studies were excluded because they were providing a formative evaluation to a proposed but not implemented instrument. This resulted in 111 articles for inclusion.

#### Summary of included studies

**Fig. 2:**
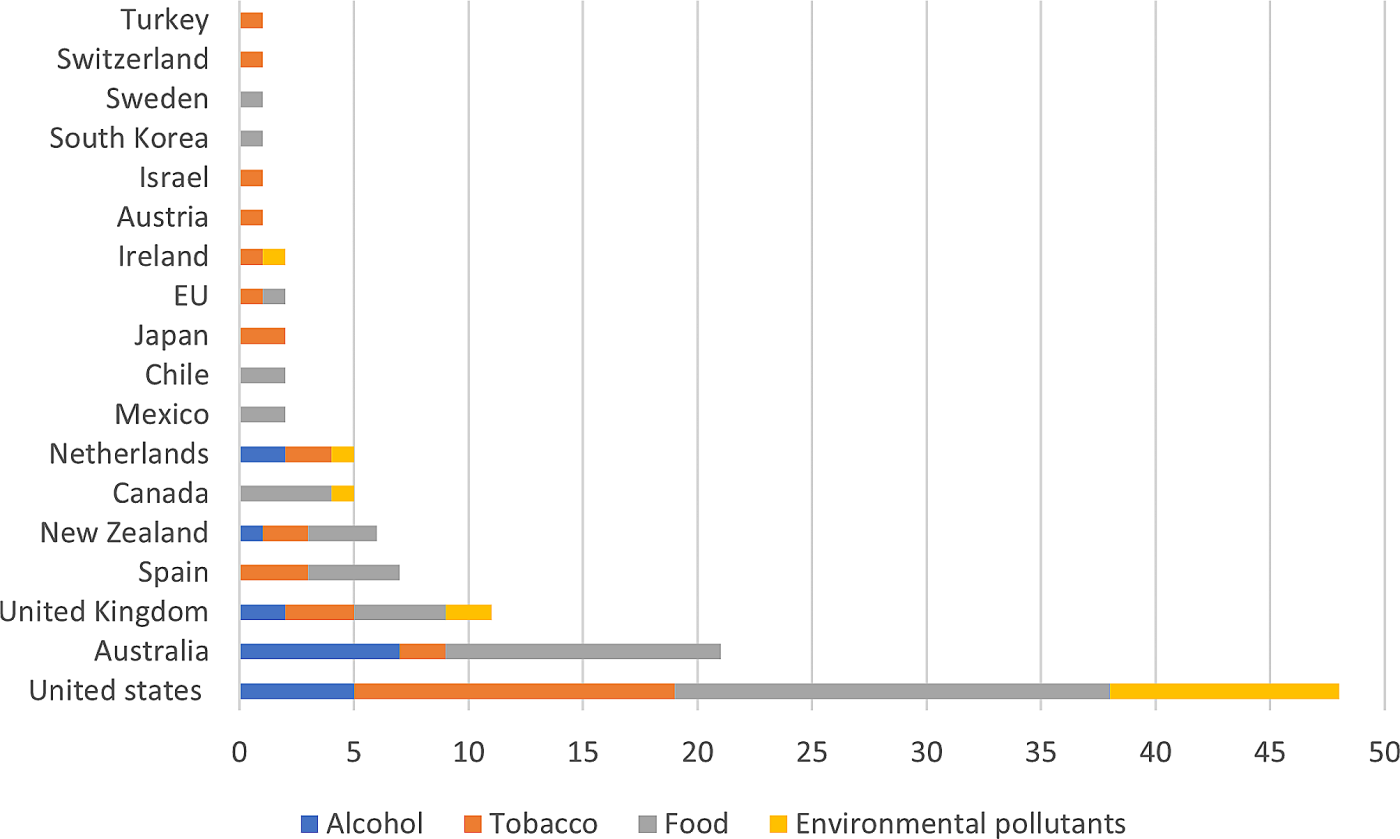
Number of included studies by country/ region and public health risk being regulated

The number of reviews and evaluations on legal and regulatory instruments has grown in recent years with the vast majority published in the past decade (Table 3). Nearly three quarters (71%) of studies focused on legal or regulatory instruments in the United States of America (USA; 42%), Australia (19%) and the United Kingdom (UK; 10%) (Fig. 2). The public health risk of concern in most studies was unhealthy food (*n* = 48) or tobacco (*n* = 34).

**Table 3:** Study characteristics

	Included studies (*n* = 111)	Proportion of studies	By risk factor			
			Alcohol	Tobacco	Food and beverages	Environmental pollutants
Year published						
*2000–2004*	4	4%	-	2	-	2
*2005–2009*	13	12%	4	8	1	1
*2010–2014*	40	36%	2	13	20	5
*2015–2020*	54	49%	11	11	27	5
Methods						
*Quantitative*	59	53%	6	22	26	6
*Cross-sectional*	21		-	10	9	2
*Multiple quant*	18		3	6	8	2
*Longitudinal*	5		1	1	2	-
*Time series analysis*	5		-	1	3	1
*Before and after*	4		-	3	1	-
*Observational*	4		1	-	3	-
*Case control*	1		-	-	-	1
*Linear modelling*	1		-	1	-	-
*Qualitative*	25	23%	4	5	14	2
*Case study – regulatory analysis*	9		1	1	6	1
*Interviews*	7		2	2	2	1
*Case study – legal analysis*	4		1	-	3	-
*Multiple qual*	4		-	2	2	-
*Content/ document analysis*	1		-	-	1	-
*Mixed*	27	24%	6	7	8	5
*Multiple Quant and qual*	9		4	3	1	1
*Cross-sectional*	5		-	2	3	-
*Case study – regulatory analysis*	4		1	-	2	1
*Case study – legal analysis*	3		-	1	1	1
*Before and after*	3		-	-	2	1
*Content/ document analysis*	3		1	1	-	1
Evaluation						
*Outcome/impact*	41	37%	4	18	15	4
*Process*	39	35%	5	10	18	6
*Process & outcome/impact*	27	24%	6	6	13	3
*Formative*	4	4%	1	-	3	-
*Economic*	0	0%	-	-	-	-
Setting						
*Single country, single instrument*	84	76%	10	30	36	9
*Single country, framework/multi-interventional*	14	13%	4	1	6	3
*Single country, multiple instruments (comparison/ individual eval. outcomes)*	8	7%	2	3	3	-
*Multiple countries, multiple instruments (comparison)*	5	5%	1	-	3	1
Country/ region						
*United states*	47	42%	5	14	19	10
*Australia*	21	19%	7	2	12	-
*United Kingdom*	11	10%	2	3	4	2
*Spain*	7	6%	-	3	4	-
*New Zealand*	6	5%	1	2	3	-
*Canada*	5	5%	-	-	4	1
*Netherlands*	5	5%	2	2	-	1
*Mexico*	2	2%	-	-	2	-
*Chile*	2	2%	-	-	2	-
*Japan*	2	2%	-	2	-	-
*EU*	2	2%	-	1	1	-
*Ireland*	2	2%	-	1	-	1
*Austria*	1	1%	-	1	-	-
*Israel*	1	1%	-	1	-	-
*South Korea*	1	1%	-	-	1	-
*Sweden*	1	1%	-	-	1	-
*Switzerland*	1	1%	-	1	-	-
*Turkey*	1	1%	-	1	-	-

Some studies evaluated multiple regulatory instruments but the majority evaluated a single regulatory instrument designed to address a single risk factor. In total 111 studies evaluated 126 instruments. Of these 126 instruments, 18 were evaluated multiple times across different studies, resulting in 90 unique legal and regulatory instruments evaluated across the included studies [see Additional file 4]. Three quarters (76%) focused on a single instrument within one country or region (such as the USA’s Family Smoking Prevention and Tobacco Control Act); 13% examined a framework or multi-interventional instrument within one country/ region (such as Philadelphia’s Lead Court and Health Code); 8% evaluated multiple comparative instruments within one country or region (such as Spain’s tobacco Law 28/2005 and its update Law 42/2010); and 4% examined multiple comparative instruments in multiple countries (such as the UK and Australia’s respective food reformulation initiatives– ‘The salt reduction program’ and the ‘Food and Health Dialogue’) (Table 3).

Just over half of the included studies (53%) used quantitative methods, one quarter (23%) were qualitative and one quarter (24%) mixed methods (Table 3). The type of evaluation undertaken was mostly outcome/ impact (37%) or process (35%). One quarter were a combined process and outcome/impact evaluation (24%), and a small number were formative evaluations (4%). When looking at the evaluation measures within the studies, the vast majority assessed implementation (*n* = 74; e.g. whether it was implemented as intended) or compliance (*n* = 47; e.g. rates of compliance), followed by changes to the built or natural environment (*n* = 43; e.g. second hand smoke or advertising exposure), reach or coverage of the instrument (*n* = 33; e.g. all advertising mediums or only certain types), acceptance (*n* = 24; e.g. support within the community), behaviour (*n* = 12; e.g. purchase behaviour or consumption rates) and socio-economic or equity considerations (*n* = 12; e.g. data stratified by demographics) (see Additional file 4). Only seven evaluations reported health outcomes (e.g. hospitalisations or mortality rates). A small number reported economic outcomes (*n* = 7; e.g. market share or consumer spending). However, there were no economic evaluations undertaken.

### Summary of regulatory design of instruments

Of the 126 regulatory instruments evaluated that focused on NCD prevention, 43% regulated unhealthy food, 30% tobacco, 16% alcohol and 12% regulated environmental pollutants, including lead, air pollution, pesticides and other chemicals (Table 4). All evaluated instruments regulated a single risk factor except one, which was an industry self-regulatory code for alcohol and tobacco advertising [21]. There is a large variety of different types of regulatory instruments, the most common being Acts, Codes, Standards, Regulations, Guidelines, Initiatives and Laws (some countries use the term ‘Law’ instead of ‘Act’). All Acts, Laws and Regulations were government regulated, all Initiatives were self-regulated or quasi-regulated, and Codes, Standards and Guidelines varied in their regulatory form, encompassing government, co- and self-regulatory approaches [see Additional File 5]. Nearly half of the included evaluations did not report on whether monitoring or enforcement of the instrument was present (47% and 45% respectively).

**Table 4:** Characteristics of regulatory instruments as reported by included studies

	Included instruments	Proportion of instruments
Risk factor		
Alcohol	20	16%
Food and beverages	54	43%
Tobacco	38	30%
Environmental pollutants	15	12%
Regulatory model		
Government regulation	90	71%
Co-regulation	4	3%
Quasi-regulation	5	4%
Self-regulation	27	21%
Mandatory or Voluntary		
Mandatory	80	64%
Voluntary	41	33%
Mixed	5	4%
Jurisdiction		
Regional (EU)	7	6%
National	89	71%
State	21	17%
Local	11	9%
Reported monitoring		
Comprehensive (i.e. proactive, transparent, independent)	29	23%
Partial (i.e. some form of monitoring in place– often reactive and/or lacking independence)	28	22%
None	10	8%
Not reported/ unclear	59	47%
Reported enforcement		
Yes	45	36%
No	24	20%
Not reported/ unclear	57	45%

### Government regulation approaches (*n* = 90)

**Fig. 3:**
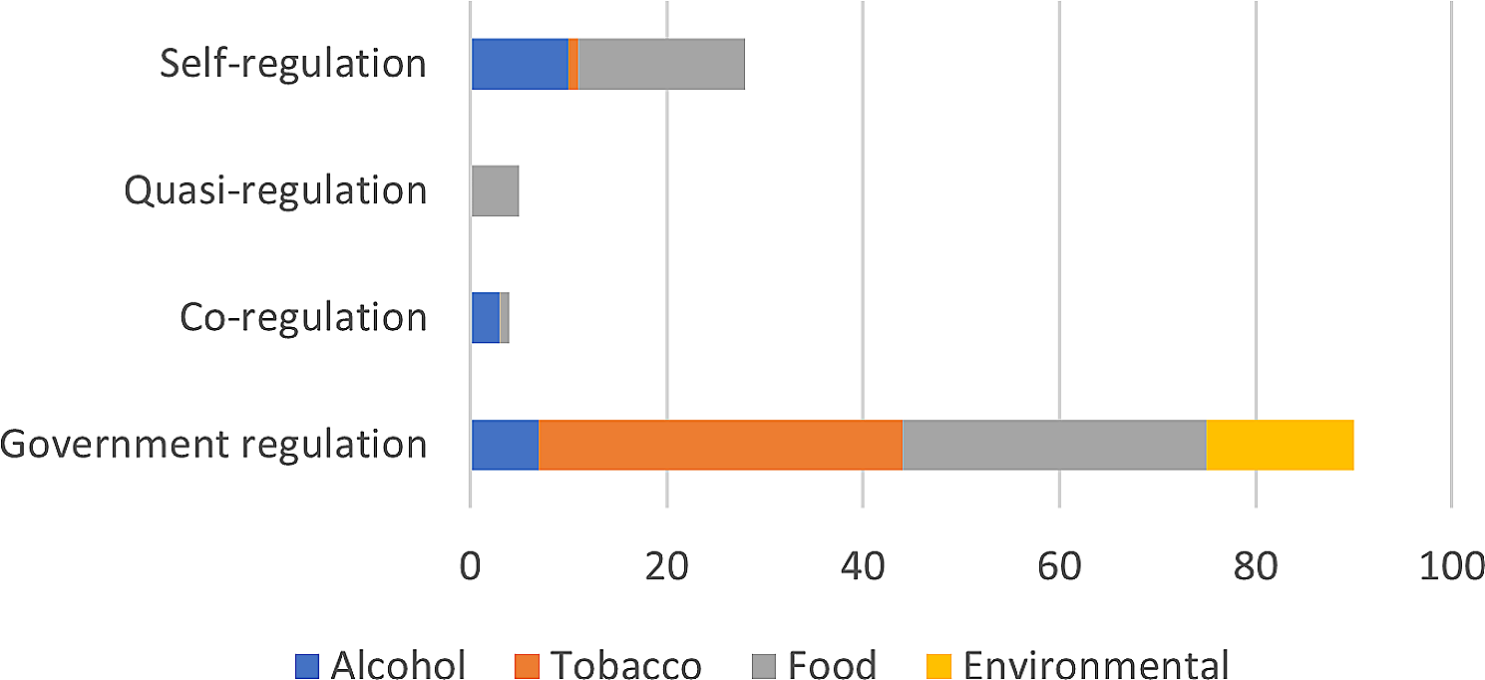
Number of evaluated instruments by regulatory form and public health risk

Government regulation was the most commonly evaluated approach for NCD prevention. Within the government regulated legal instruments, the majority targeted tobacco (*n* = 37) and unhealthy food (*n* = 31), followed by environmental pollutants (*n* = 15) and alcohol (*n* = 7) (Fig. 3). The vast majority were mandatory (87%; *n* = 78) [22–90], and a small proportion took a voluntary (9%; *n* = 8; [80, 89, 91–95]) or mixed approach (4%; *n* = 4; [96–99]). Monitoring and enforcement was largely unreported in the literature (52% and 50% respectively). Of those that did report on monitoring (*n* = 43), 39 had some form of monitoring present (comprehensive [23, 26, 31–34, 39, 41, 42, 45–47, 56, 59, 62, 64, 66, 70, 69, 78, 79, 88, 93, 97, 100, 101]; partial [24, 25, 50, 52, 60, 67, 71, 74, 75, 80, 83, 98]), and four simply stated that there was no monitoring mechanism in place [43, 51, 54, 92]. Of those that did report on enforcement (*n* = 46), 36 detailed some form of enforcement mechanism, and 9 state that no enforcement occurred [43, 49, 51, 90–92, 99]. The policy area they were regulating was largely availability (*n* = 69), with some focusing on education/ training/ reporting (*n* = 17), labelling (*n* = 16), and marketing (*n* = 11). Only two regulated price.

### Co-regulation approaches (*n* = 4)

There were few co-regulatory approaches evaluated in the included studies, with one examining a single instrument [102] and three evaluating multi-interventional frameworks [103–105]. The instruments regulated alcohol [102, 103, 105] and unhealthy food [104] (Fig. 3). Three took a co-regulatory approach with industry on food or alcohol marketing and one was a co-regulatory approach with community regarding alcohol availability [105]. The design and implementation varied taking mandatory [103, 104], voluntary [102] and mixed approaches [105]. Where evaluations described monitoring it was comprehensive [105] or non-existent [102]. Enforcement was reported in two studies but the authors described them as weak [102, 103].

### Quasi-regulation (*n* = 5)

Five evaluations within three included studies (two multi-country comparisons and one single country) were conducted on three different quasi-regulatory instruments. The reported design and implementation was very similar. All targeted unhealthy food, specifically food reformulation, all were voluntary, and none were enforced [5, 106, 107]. Four evaluations reported some form of monitoring [5, 107] and one did not report on monitoring [106].

### Self-regulation (*n* = 27)

The self-regulatory instruments evaluated in the included studies focused on food (*n* = 17), alcohol (*n* = 9), and tobacco (*n* = 1) (Fig. 3). Most self-regulated marketing [6, 21, 87, 103, 104, 108–125] with two targeting alcohol labelling [126, 127] and another targeting alcohol availability and education/ training (responsible service) [54]. All were voluntary. The approach to monitoring and enforcement was varied. Twelve studies did not discuss or provide details of monitoring [87, 114, 117–119, 121–123, 128] or enforcement [104, 110, 111, 117–119, 121–123, 128]. Five reported that the legal instrument had no monitoring or enforcement [21, 54, 109, 126, 127], and another five reported no enforcement but some monitoring [87, 108, 113–115]. In total, thirteen of the self-regulatory instruments reported some form of non-comprehensive monitoring (partial) that was either complaints based [103, 108, 113, 115, 116, 120, 124, 125] or in the form of annual reporting [6, 104, 110–112, 115, 116]. Seven instruments had some level of enforcement, however, it was not occurring in practice [103, 112] or was very minimal such as requesting the removal of non-compliant advertisements [124, 125], publishing a list of non-compliant businesses (a ‘name and shame’ approach) [6, 116] or exclusion from the code [120]. None were conducted by an independent body.

### Reported effectiveness

The effectiveness of the regulatory instrument was reported on in all included papers. This ranged from authors reporting effectiveness of implementation against a regulatory framework, to clearly reported quantitative measures of health or behavioural impacts. The way that effectiveness was considered varied by evaluation type. Most looked at more than one variable. An additional file provides the number of effective instruments by evaluation type and outcome measure [see Additional file 6].

Of the instruments reviewed (*n* = 126) in the included studies, half reported some level of effectiveness (49%; *n* = 46 yes, *n* = 15 partial), and the other half (51%; *n* = 65) were ineffective at achieving the measured outcome.

### Reported effectiveness by regulatory form

**Fig. 4:**
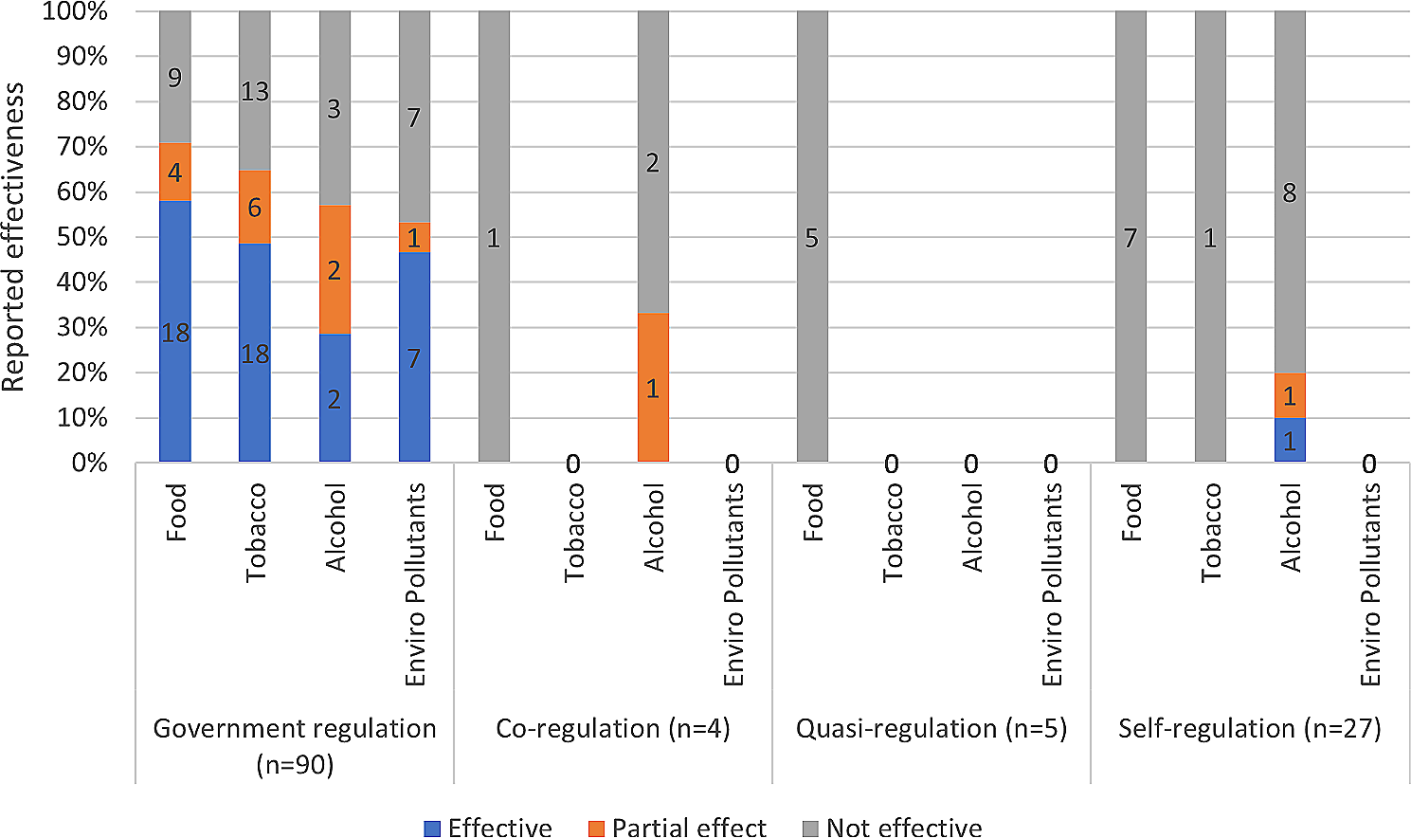
Reported effectiveness of regulatory instruments by regulatory form and risk factor

When looking at effectiveness by regulatory approach, 58 of the 90 government regulated instruments were reported by study authors to have some level of impact (Fig. 4). This is compared to only 1 of the 4 co-regulated instruments and 2 of the 27 self-regulated instruments that were reported to have some level of impact. No quasi-regulated instruments were reported to be effective. The ineffective instruments were considered by authors to be largely ineffective due to implementation barriers, non-compliance and limited reach or coverage. Self-regulatory instruments were also found to be largely ineffective at changing outcomes to the built and natural environment. An additional file provides more detail on the reported effectiveness of regulatory instruments by regulatory form and evaluation outcome measures [see Additional file 7].

The reported effectiveness of regulatory instruments by public health risk factor also varied. Alcohol and food were more likely to be self-regulated and more frequently reported to be ineffective compared to regulatory instruments for tobacco and environmental pollutants (Fig. 4). However, when food regulatory instruments were government regulated (*n* = 31), they were more likely to be reported as effective (*n* = 22; completely and partially). Government regulated instruments for tobacco (*n* = 24), alcohol (*n* = 4) and environmental pollutants (*n* = 8) were also found to have some level of impact majority of the time (65%, 57% and 53% respectively) (Fig. 4).

## Discussion

Public health law evaluations have seen considerable growth in numbers since 2000, with the largest numbers in the past decade. This aligns with the demand for evidence-based policy making and policy makers’ need to better evaluate and understand laws and regulations that impact public health. There is a strong culture of systematic reviews of regulatory interventions [129–133]. However, our scoping review shows there remains a disconnect between scientific evaluation and conventional legal scholarship. There is a significant amount of detail lacking in scientific evaluations regarding the design and implementation of regulatory instruments. Over 100 evaluations were excluded because they did not provide sufficient detail of the regulatory instrument. Evaluations also lacked details regarding whether OECD best practice regulation was occurring. These details are important for understanding what design and implementation elements can better promote effective public health interventions.

### Regulatory design and effectiveness

There is a large range of regulatory approaches and instruments utilised for the prevention of NCDs. Our review found that government regulation was the most commonly evaluated approach and the most likely to be effective. This is compared to self-regulation and quasi-regulation which were almost always reported to be ineffective. This was particularly evident for unhealthy food, which was reported highly effective when government regulated and not effective when self- or quasi-regulated. The only effective self-regulatory instrument was designed and administered by a public health organisation, independent of industry, with the purpose of countering the alcohol industry’s ineffective voluntary scheme [116]. Another was reported to be partially effective, however the authors raised questions about the efficacy, as despite there being high compliance among signatories to the industry developed voluntary guidelines, the guidelines themselves did not align with the Attorney General’s recommendations [122]. 

There were few quasi-regulation instruments evaluated. This may be because this is not a well-defined or commonly used governance mechanism. Quasi-regulation typically features and has been well defined in the context of food policy [5, 6] but is often used interchangeably in broader regulatory contexts with other approaches [134]. However, of those included in our review, none were effective. For example, Magnusson & Reeve (2015) compared voluntary quasi-regulatory food reformulation instruments in Australia and the UK and reported both instruments to be ineffective. The evaluation of the Australian instrument found there had not been any nutritional improvement in the target products, there was incomplete coverage of industry with limited participation, and limitations in governance with no monitoring and self-reported compliance. The evaluation of the UK instrument found the salt reduction target had not been achieved, there was inconsistent coverage and industry participation, and a lack incentives and disincentives to encourage participating.

The results for co-regulatory approaches were unclear due to the small number identified in the included studies. Co-regulation is thought to be of value as it reduces the administrative burden and associated costs on government, however, due to the close relationships required between industry and government there is a higher risk of regulatory capture [134–137]. Much of the theoretical literature discusses best practice principles of co-regulation, including transparency, accountability and concern for reputational risk [136, 138]. Yet, there appears to be a lack of empirical evaluations of co-regulatory approaches to NCD prevention making it difficult to draw conclusions regarding effectiveness in practice. More empirical evaluations of co-regulatory approaches could help to better understand the effectiveness of this model.

Government regulation was largely mandatory with a small proportion taking a voluntary or mixed approach. Voluntary and mixed government regulated approaches were reported to be effective half the time (50%) compared to majority of the time (87%) for mandated government regulation. Co-regulation utilised voluntary and mandatory approaches, quasi-regulation and self-regulation were all voluntary. Given the reported in-effectiveness of quasi- and self-regulation, and the mixed results for voluntary government and co-regulation, it appears that voluntary approaches are ineffective at achieving the desired outcome. For example, an evaluation of voluntary government nutrition guidelines for Mexican schools found the voluntary nature and lack of monitoring and compliance demonstrated poor uptake and low compliance, resulting in a lack of effectiveness [92]. Another evaluation of voluntary food reformulation in the UK and USA found a disproportionate level of influence on the governing bodies by industry, and that there was a lack of specific and time-bound commitments, lack of participation across the sector, inadequate monitoring mechanisms and lack of enforcement options [107]. Voluntary approaches are promoted by industry groups in an attempt to avoid explicit government regulation and as a mechanism to build trusted relationships with governments [139, 140]. There is a wealth of literature on the ineffectiveness of voluntary approaches in public health law [129, 141–144]. Voluntary regulation has also been criticised in areas not included in this review including pharmaceuticals [145] and food safety [136] due to low uptake and limited accountability mechanisms.

The overall effectiveness of instruments by risk factor indicated that food and alcohol regulation were reported to be ineffective more often. However, when analysing studies by regulatory form, food and alcohol were more likely to be self-regulated or quasi-regulated compared to tobacco and environmental pollutants. This likely impacts their overall effectiveness. Our results indicate when food and alcohol are government regulated they appear to be more effective. This was particularly apparent for food regulation. Food laws and regulation are not necessarily less effective than tobacco but rather they are less effective due to their design and governance mechanisms being largely quasi- and self-regulated, and lacking independence [142, 146]. These regulatory approaches also lack transparency and accountability.

It is likely that food and alcohol are more frequently self- or quasi-regulated as they do not have the same extensive history of advocacy and research, nor the international laws that govern tobacco and environmental pollutants. International treaties such as the World Health Organization Framework Convention on Tobacco Control (2003), the United Nations Environment Programme Stockholm Convention on Persistent Organic Pollutants (2001) and the International Labor Organization Asbestos Convention (1990) are global treaties to protect human health that require Parties to take measures to prevent and eliminate exposure to harmful products. They have played an instrumental role in shaping local government laws and evolving the regulation of tobacco and environmental pollutants beyond voluntary approaches. Despite decades of evidence on the risks of unhealthy food and beverages including alcohol, some have suggested that these unhealthy commodities remain largely self-regulated or governed by voluntary agreements because these industries have successfully positioned themselves as part of the solution and have learnt from the ‘mistakes’ of the tobacco industry [146, 147]. Additionally, the approach taken is ultimately a decision of government policy and given the sheer effort and decades of research and advocacy needed to implement government regulation, it is likely that these risk factors have been secondary to tobacco and environmental pollutants.

The argument for regulating tobacco is also much clearer in that there is no health benefit to tobacco consumption. For food there is the claim that there may be some nutritional value and that eating small amounts of unhealthy food or drinking small amounts of alcohol are not as damaging compared to tobacco. Therefore, there may not be the political or public will to advocate for mandatory government regulation. The barrier to mandatory food regulation may be even higher as the health claims for alcohol have in recent years been largely rejected by evidence [148, 149]. This has brought about a perception of complexity when it comes to regulating unhealthy food and beverages. This perception may stem from concerns that such integration could introduce unfamiliar complexities, potentially leading to challenges in the implementation and enforcement, and that food is seen as a personal choice that doesn’t inherently harm others the way that tobacco and alcohol can. Additionally, fast-food outlets contribute to local economies by providing employment opportunities and generating revenue. Moreover, the challenges associated with obesity and dietary problems are recognised as multifaceted and extending beyond single factors that can be regulated (e.g. density of outlets) so policymakers are unclear about the potential effectiveness of these interventions. This has often led policy makers to, rather than regulating density, direct efforts towards increasing public awareness, promoting healthier food choices, empowering individuals to make informed dietary choices, and encouraging the food industry to offer healthier alternatives.

Public health researchers have previously stated that there is an inherent conflict of interest in allowing unhealthy commodity industries regulatory control over public health matters [141, 142]. The OECD principles of best practice regulation also prioritise independence, accountability, transparency, preventing undue influence and maintaining trust [10]. Yet, our review shows that regulatory approaches that do not align with these principles remain common practice. There is an industry preference for voluntary, self-regulatory or partnership approaches, providing businesses with more control and the ability to avoid impact on successful business models by utilising less effective regulatory approaches [142, 150]. This renders co-, quasi- and self-regulation problematic from a public health perspective and reinforces concerns around issues of regulatory capture with partnership approaches.

Interestingly the policy area being regulated also varied significantly by the different regulatory form and governance mechanisms. Government regulation (*n* = 90) focused largely on availability (78%); co-regulation focused mainly on marketing (75%); quasi-regulation focused exclusively on reformulation (100%); and self-regulation focused largely on marketing (88%). Again this is a product of policy decisions and the political economy. It could also be that availability of harmful and unhealthy products has been seen to be more in the public interest and therefore requiring explicit regulation for consumer protection [151]. While marketing and reformulation may be perceived by governments to be more amendable to co-ordination and co-operation, principles that could be achieved via ‘win-win’ partnerships through ostensibly softer regulatory approaches such as co-, quasi- or self-regulation [152]. Ideology also plays a role, with conservative and centrist governments viewing food and beverage choices as personal and individualistic, and therefore restricting marketing via government regulation is not seen as a valid solution [153]. However, as previously mentioned partnership and self-regulatory approaches are susceptible to regulatory capture, conflicts of interest and limited transparency and accountability; particularly as there are limited sanctions on industry players who continue to push back the boundaries on these regulations. Additionally, marketing regulation has become more complex with the rapid growth of new marketing techniques and the huge influence of digital marketing leaving many governments uncertain in how to regulate [154]. There is however examples of government marketing regulation such as new laws in Australia for influencer marketing of supplements and health care products [155], and in the UK for digital junk food marketing [156], which could be evaluated in future to support the public interest argument in other jurisdictions.

### Quality and extent of information reported within the included studies

It was unclear whether the presence of monitoring or enforcement impacted the effectiveness of a regulatory instrument due to large inconsistencies in reporting. Approximately half of the included evaluations and reviews did not provide detail of whether monitoring or enforcement was present. This is critical design information that can help better understand effectiveness of interventions. Other literature has highlighted the importance of independent monitoring and enforcement as key design features for effective regulation. A comparative analysis of regulatory governance conditions identified comprehensive monitoring as a necessary (or required) condition for positive nutrition outcomes in 94% of cases [157]. They also identified a combination of conditions that when used together are sufficient at providing positive outcomes: absence of high industry involvement, strict regulatory design, best practice design and comprehensive monitoring and enforcement (96% of cases); or absence of high industry involvement, best practice design and comprehensive monitoring (82% of cases). It will be difficult to draw associations through future systematic reviews if evaluations are not reporting vital information regarding monitoring and enforcement. Included studies also infrequently reported on duration of implementation, important factors for assessing effectiveness.

Research in this area should provide more detail of the design and implementation features of the legal intervention they are evaluating. This is critical information to policy makers who may seek to replicate interventions in their own jurisdictions or learn lessons from ineffective attempts. It is also important for understanding the history of the law in question and how it may have evolved overtime following evaluations and government reviews.

There was also a spectrum of what was considered effective by authors and different outcome measures and methodologies used. While this scoping review was not intended as a rigorous quantitative synthesis of the rigor of legal evaluations, it does describe and highlight the diversity of the public health law evaluation literature. For example, Yorifuji 2011 conducted an outcome/ impact evaluation of a mandatory government regulated tobacco law using quantitative methods to measure compliance and correlations with behaviour (decreases in smoking rates) and health (decreases in lung cancer) outcomes. They found the instrument to be effective with increased compliance associated with decreased prevalence of tobacco smoking, and decreased tobacco smoking associated with decreased cancer mortality. While Hadfield 2015 undertook a process and outcome/ impact evaluation using mixed methods to examine implementation and compliance with legislation (mandatory government regulated) and a supporting corporate social responsibility initiative (voluntary self-regulated) for liquor licence premises. They found both the voluntary and statutory frameworks were ineffective and failing to drive good practice because the structures for monitoring and enforcing the legislation were expensive, resource intensive and required evidence that was difficult to obtain, and the self-regulated instrument was not being consistently adopted, lacked clear targets and had high rates of non-compliance.

### Strengths of our review

To our knowledge this is the first study to map the diversity of regulatory evaluation literature and draw linkages between design features and reported effectiveness across a range of public health risk factors. We utilised a comprehensive search strategy across health and legal databases considering multiple risk domains and all legal instruments across OECD countries. The strength of our findings is a testament to this comprehensive approach, focusing on what does and does not work, thus providing a good distribution of effective and ineffective models. The results of this paper may be known anecdotally but this paper empirically reports it.

### Limitations of our review

The assessment of effectiveness was based on what the authors of the papers reported. We did not independently verify the claims of each paper. Assessment of monitoring and enforcement was based on whether it was reported to be a design feature, not whether monitoring or enforcement was actually working in practice. Due to the size and nature of this scoping review it was not possible to analyse the effectiveness of monitoring or enforcement specifically. However, it is worth noting that some evaluations reporting monitoring or enforcement stated they were limited in effectiveness.

Another limitation is the small sample of co-regulated and quasi-regulated instruments. While quasi-regulation is less common, co-regulation is frequently used in public health and it is surprising only four regulatory instruments were identified in the included studies. There also appears to be some inconsistencies in how authors categorised legal instruments by regulatory form. Three quarters of evaluations examined a single regulatory instrument. While this is common, they can be difficult to evaluate if they sit as part of a framework as it is hard to isolate what components are effective.

There were few included studies that focused on pricing regulations. There is extensive literature on taxation and other pricing policies that has not been captured by our scoping review [158]. It is possible that our search strategy did not capture some of this literature that was published before the year 2000. There were also numerous studies on taxation that did not pass full text review as they had study designs outside our scope or they did not provide enough detail of the regulatory instrument being evaluated.

It is also likely that many evaluations may not have made it into peer-review literature due to weak study designs or lack of interest in submitting them to peer-reviewed journals (e.g. government evaluations). Our results found there may be limitations with some of the included study designs. Implementation was the major focus of the included evaluations and very few focused on health or economic outcomes, with no cost-benefit or economic evaluations identified. Stronger study design and the use of rigorous quantitative methods such as interrupted time series analyses should be encouraged to support qualitative implementation assessments. Regulatory instruments are regularly reviewed by government through internal or parliamentary process. It was not within our scope to include these in this review. Some regulations may have been updated following their evaluations, while others may not have. This scoping review is not able to look at changes to regulations following evaluations.

Last, nearly half (42%) of the evaluations are coming from the USA, a high-income country with a unique system of governance. This may also make the transferability of some findings difficult. The field of empirical public health law has originated from the USA, likely contributing to the high number of publications from that country. Many low and middle income countries (LMICs) were also excluded from this review due to the focus on the OECD. Another limitation of our scoping review is its focus on English only publications. We acknowledge that there is work occurring on NCD prevention regulation within countries outside of the OECD and that evaluations are occurring in non-English speaking languages, which have not been captured by our search and may offer lessons for future research.

Our search strategy yielded few studies on pricing when we know there is extensive literature in this area.

### Implications for future research

As we focused on the OECD, future work could look at countries outside of this group, in particular LMICs which are not well represented in our scoping review. For example, countries in Central and South America have shown leadership in new food and beverage regulation, such as Mexico’s sugar-sweetened beverage tax, which fell outside the scope of our review. LMICs may also provide more evidence on co-regulatory approaches, as they tend to have more limited regulatory capacity, rendering explicit government regulation unfeasible in some contexts. It will be interesting to see what approaches are taken in other contexts and how public health law evaluations are occurring in LMICs. More evaluations on co-regulatory approaches in countries of varying economic contexts would be beneficial.

The Hein Online legal database is not user friendly for systematic review searches in the way that medical and social science databases are. This is likely because systematic reviews are not a methodology used in legal scholarship. However, as the area of empirical legal evaluations grows this may become more common practice. Researchers wishing to conduct similar work in public health law should be aware of the limitations of legal databases.

The included evaluations in this review largely focused on the regulation of unhealthy food and tobacco. More evaluations could be conducted on alcohol and environmental pollutants (including asbestos and chemicals) as there are many lessons to learn from the regulation of these products that may currently be missing from the literature. There were also many studies on other public health risk areas such as pharmaceuticals, food safety and road safety, which were excluded from this review but could feature in future scoping or systematic reviews. Future systematic reviews could also look to draw associations between design and effectiveness, which was not able to be done through this scoping review.

Moving forward scientific research should provide details of the law being evaluated including whether it is voluntary or mandatory and whether monitoring or enforcement is in place. This can sometimes be difficult when publishing in scientific journals with limited word counts, however, this is important evidence that needs to be reported for better understanding and potential replication of the instrument being evaluated.

## Conclusion

Public health law research is a growing field with some disconnect between scientific research and legal scholarship. This scoping review maps the diversity of regulatory evaluation literature and demonstrates the need for public health law research to better bridge this gap in order to draw linkages between design features and effectiveness of legal interventions. Governments should continue to adopt independent, transparent and evidence-based regulations and support their evaluation. Government regulation is the most commonly used and most effective regulatory approach in the public health law literature. Food and alcohol sectors are more likely to adopt self- or quasi-regulation and are frequently reported as ineffective. More work should be done to support government regulation in these areas. Diverse evidence will be required including research focused on implementation barriers and enablers, health and economic outcomes. To help move towards more government regulation of public health risk factors, future research should ensure it includes enough detailed information of the regulatory design so other jurisdictions can learn from successes and failures.

### Electronic supplementary material

Below is the link to the electronic supplementary material.

Additional file 1

Additional file 2

Additional file 3

Additional file 4

Additional file 5

Additional file 6

Additional file 7

## Data Availability

The datasets generated and/or analysed during the current study are not publicly available due to ongoing analyses for future publications but are available from the corresponding author on reasonable request.
