# Supplementary material for: Legal and regulatory instruments for NCD prevention: a scoping review and descriptive analysis of evaluations in OECD countries
Source: BMC Public Health. 2024 Feb 29;24:641. doi: 10.1186/s12889-024-18053-4 (PMC10903077; doi:10.1186/s12889-024-18053-4)
Supplement: Supplementary file 1 — Additional file 1 [file 12889_2024_18053_MOESM1_ESM.docx]

*Additional file 7: Reported effectiveness of regulatory instruments by regulatory form and evaluation outcome measures*

| Regulatory form | Reported effectiveness | Evaluation outcome measures* | | | | | | | | |
| --- | --- | --- | --- | --- | --- | --- | --- | --- | --- | --- |
|  |  | **Implementation** | **Acceptance** | **Reach** | **Health** | **Behaviour** | **Environment** | **Compliance** | **Socio-economic** | **Economic** |
| Government regulated (n=90) | Yes (n=45; 50%) | 22 | 9 | 8 | 4 | 7 | 15 | 14 | 6 | 4 |
|  | Partial (n=14; 16%) | 10 | 3 | 2 | 1 | 2 | 4 | 4 | 1 | 0 |
|  | No (n=31; 34%) | 21 | 9 | 7 | 2 | 3 | 5 | 13 | 3 | 2 |
| Co-regulated (n=4) | Yes (n=0) | - | - | - | - | - | - | - | - | - |
|  | Partial (n=1; 25%) | 1 | 1 | - | - | - | - | - | - | - |
|  | No (n=3; 75%) | - | 1 | - | - | - | 3 | 2 | - | - |
| Quasi-regulated (n=5) | Yes (n=0) | - | - | - | - | - | - | - | - | - |
|  | Partial (n=0) | - | - | - | - | - | - | - | - | - |
|  | No (n=5; 100%) | 5 | - | 4 | - | - | - | - | - | - |
| Self-regulated (n=27) | Yes (n=1; 4%) | 1 | - | - | - | - | - | - | - | - |
|  | Partial (n=1; 4%) | 1 | - | - | - | - | - | - | - | - |
|  | No (n=25; 93%) | 14 | 2 | 11 | - | - | 15 | 12 | 2 | 1 |

*values add to more than total as evaluations had multiple outcome measures. See data dictionary in supplementary files for definitions and examples of measures
