# Supplementary material for: Legal and regulatory instruments for NCD prevention: a scoping review and descriptive analysis of evaluations in OECD countries
Source: BMC Public Health. 2024 Feb 29;24:641. doi: 10.1186/s12889-024-18053-4 (PMC10903077; doi:10.1186/s12889-024-18053-4)
Supplement: Supplementary file 2 — Additional file 2 [file 12889_2024_18053_MOESM2_ESM.docx]

Additional file 3: Data dictionary supporting extraction and analysis

| **Term** | | **Definition or example** |
| --- | --- | --- |
| Evaluation | Outcome/impact | Evaluation steps focusing on achieving certain goals. |
|  | Process | Evaluation steps focusing on implementation, acceptance and reach. Helps to understand how and/or why the legal instrument was adopted in a particular way, and was or was not effective. |
|  | Process & outcome/impact | Evaluation steps focusing on how and/or why the legal instrument was adopted, and how is achieved certain goals. |
|  | Formative | Evaluation steps before the launch or implementation of the legal instrument. |
|  | Economic | Evaluation steps focusing on identifying, measuring, valuing and comparing costs and consequences of the legal intervention. |
| Evaluation measure/ outcome | Health | Change in health status or biological indicator e.g. disease prevalence, mortality |
|  | Behaviour | e.g. purchasing, smoking rates |
|  | Lived or built environment | e.g. advertising exposure, reformulation |
|  | Compliance | e.g. rates of compliance |
|  | socio-economic considerations | e.g. reports outcomes for different populations |
|  | Economic | e.g. revenue, market share |
|  | Implementation | e.g. as intended |
|  | Acceptance | e.g. support from public, industry, public service |
|  | Reach | e.g. coverage, comprehensiveness |
| Setting | Single country, single instrument | The focus is on one specific legal instrument in one country. |
|  | Single country, framework/multi-interventional | The focus is on multiple related legal instruments as part of a framework in one country. |
|  | Single country, multiple instruments (comparison/ individual eval. outcomes) | The focus is on comparing more than one legal instrument within the same country. |
|  | Multiple countries, multiple instruments (comparison) | The focus is on comparing more than one legal instrument within multiple countries. |
| Mandatory or voluntary | Mandatory | The regulatory instrument is mandated and all relevant stakeholders must comply with it |
|  | Voluntary | The regulatory instrument is optional and stakeholders can choose to adopt it |
|  | Mixed | Components of the regulatory instrument are mandatory and components are voluntary. |
| Reported monitoring | Comprehensive | The authors report monitoring is occurring by an independent body, is proactive and transparent. |
|  | Partial | The authors report there is some form of monitoring in place, which may be reactive and/or lacking independence. |
|  | None | The authors report there is no form of monitoring present. |
|  | Not reported/ unclear | The authors do not report on or provide details of whether monitoring is present. |
| Reported enforcement | Yes | The authors report there is some form of enforcement of the regulatory instrument present. This does not assess whether it is fit-for-purpose or effective in practice. |
|  | No | The authors report there is no enforcement of the regulatory instrument. |
|  | Not reported/ unclear | The authors do not report on or provide details of whether enforcement is present. |
| Reported effectiveness | Yes | The authors report that the legal instrument has achieved the intended outcome. |
|  | Partial | The authors report that the legal instrument has partially achieved the intended outcome. |
|  | No | The authors report the legal instrument has not achieved the intended outcome, unclear results or a negative effect. |
