# Supplementary material for: Legal and regulatory instruments for NCD prevention: a scoping review and descriptive analysis of evaluations in OECD countries
Source: BMC Public Health. 2024 Feb 29;24:641. doi: 10.1186/s12889-024-18053-4 (PMC10903077; doi:10.1186/s12889-024-18053-4)
Supplement: Supplementary file 3 — Additional file 3 [file 12889_2024_18053_MOESM3_ESM.docx]

Additional file 6: Number of effective instruments by evaluation type and outcome measure

| **Evaluation type** | **Outcome measure** | **Effective** | **Partial effectiveness** | **Not effective** |
| --- | --- | --- | --- | --- |
| **Process (46 instruments)** | Implementation | 13 | 8 | 23 |
|  | Acceptance | 4 | 4 | 5 |
|  | Reach | 5 | 2 | 13 |
|  | *total* | *13* | *8* | *25* |
| **Process & outcome/ impact (31 instruments)** | Implementation | 9 | 3 | 15 |
|  | Acceptance | 5 |  | 6 |
|  | Reach | 1 |  | 6 |
|  | Health | 1 |  |  |
|  | Behaviour |  |  |  |
|  | Environment | 5 | 2 | 9 |
|  | Compliance | 6 | 2 | 11 |
|  | Socio-economic & equity considerations |  |  | 3 |
|  | Economic | 3 |  | 1 |
|  | *total* | *11* | *3* | *17* |
| **Outcome/ impact (45 instruments)** | Health | 3 | 1 | 2 |
|  | Behaviour | 6 | 2 | 4 |
|  | Environment | 9 | 3 | 15 |
|  | Compliance | 8 | 3 | 16 |
|  | Socio-economic & equity considerations | 5 | 1 | 1 |
|  | Economic | 1 |  | 2 |
|  | *total* | *18* | *5* | *21* |
| **Formative (4 instruments** | Implementation | 2 |  |  |
|  | Reach | 3 |  | 1 |
|  | Socio-economic | 1 |  | 1 |
|  | *total* | *3* |  | *1* |
