# Supplementary material for: Legal and regulatory instruments for NCD prevention: a scoping review and descriptive analysis of evaluations in OECD countries
Source: BMC Public Health. 2024 Feb 29;24:641. doi: 10.1186/s12889-024-18053-4 (PMC10903077; doi:10.1186/s12889-024-18053-4)
Supplement: Supplementary file 4 — Additional file 4 [file 12889_2024_18053_MOESM4_ESM.docx]

*Additional file 5: Type of regulatory instruments evaluated and their regulatory form*

| Type | No. included | Proportion of instruments | Regulatory form |
| --- | --- | --- | --- |
| Act | 38 | 30% | G |
| Code | 25 | 20% | G, C, S |
| Standard | 10 | 8% | G, C, S |
| Regulation | 9 | 7% | G |
| Guideline | 6 | 5% | G, S |
| Initiative | 7 | 6% | Q, S |
| Law | 7 | 6% | G |
| Ordinance | 5 | 4% | G |
| Scheme | 5 | 4% | G, S |
| Directive | 5 | 4% | G |
| Program | 4 | 3% | G, Q, S |
| Dialogue | 2 | 2% | Q |
| Executive Order | 1 | 1% | G |
| Grant | 1 | 1% | G |
| Bylaw | 1 | 1% | G |
| Certification | 1 | 1% | G |
| Pledge | 1 | 1% | S |
| Practice Note | 1 | 1% | G |
| Rule | 1 | 1% | G |
| Strategy | 1 | 1% | G |
| Trust | 1 | 1% | C |
| Decree | 1 | 1% | G |
| Specialist court | 1 | 1% | G |

** G= government regulation, C= co-regulation, Q= quasi-regulation, S= self-regulation*
