# Supplementary material for: Legal and regulatory instruments for NCD prevention: a scoping review and descriptive analysis of evaluations in OECD countries
Source: BMC Public Health. 2024 Feb 29;24:641. doi: 10.1186/s12889-024-18053-4 (PMC10903077; doi:10.1186/s12889-024-18053-4)
Supplement: Supplementary file 5 — Additional file 5 [file 12889_2024_18053_MOESM5_ESM.docx]

Additional file 4: Summary of included studies and data extracted

| **Author, year** | **Study characteristics** | | | | | **Legal characteristics** | | | | | | **Reported outcomes** | | |
| --- | --- | --- | --- | --- | --- | --- | --- | --- | --- | --- | --- | --- | --- | --- |
|  | **Country/ region** | **Methods** | **Method type** | **Evaluation type** | **Evaluation measure** | **Title** | **Type** | **Regulatory model** | **Voluntary/ mandatory** | **Public health risk** | **Policy type** | **Monitor-ing** | **Enforce-ment** | **Reported effective-ness** |
| Alpert 2018 | United States | Quantitative | Cross-sectional | Outcome/ impact | Environment, behaviour, economic | Family Smoking Prevention and Tobacco Control Act 2009 | Act | Government | Mandatory | Tobacco | Marketing, labelling | Comprehensive | Present | Not effective |
| Arcury-Quandt 2011 | United States | Qualitative | Interviews | Outcome/ impact | Behaviour, Compliance, socio-economic | Workplace Right-to-Know Act | Act | Government | Mandatory | Environmental pollutant | Labelling, education/ training, reporting | Partial | Not reported | Not effective |
| Barnes 2018 | United Kingdom | Qualita-tive | Case study (regulatory analysis) | Process | Implementation | Ambient Air Quality Directive (2008/50/EC) | Directive | Government | Mandatory | Environmental pollutant | Availability | Comprehensive | Not reported | Not effective |
| Baron-Epel 2012 | Israel | Qualitative | Interviews | Process & outcome/ impact | Implementation, acceptance, compliance | The Prevention of Smoking and Exposure to Smoking in Public Places Law 1983 | Law | Government | Mandatory | Tobacco | Availability | Partial | Present | Not effective |
| Barquera 2018 | Mexico | Quantitative | Cross-sectional, observational | Outcome/ impact | compliance, environment | Publicidad de alimentos y bebidas dirigidas a al población infantil | Code | Self-regulation | Voluntary | Food | Marketing | Absent | Absent | Not effective |
| Becher 2019 | Australia & New Zealand | Qualitative | Case study (legal analysis) | Process & outcome/ impact | Implementation, reach, socio-econmic considerations | Health Star Rating Scheme | Scheme | Self-regulation | Voluntary | Food | Labelling | Absent | Absent | Not effective |
| Benjamin-Neelon 2017 | United States | Quantitative | Cross-sectional | Process | Implementation, reach | Caring for Our Children: National Health and Safety Performance Standards | Standard | Government | Voluntary | Food | Education/ training, availability | Not reported | Absent | Partial effect |
|  |  |  |  |  | Implementation, reach | Guidelines for Out-of-Home Child Care Programs. | Guideline | Government | Voluntary | Food | Education/ training, availability | Not reported | Absent | Effective |
| Bruemmer 2012 | United States | Quantitative | Observational | Outcome/ impact | environment | King County Board of Health restaurant menu labeling regulation | Regulation | Government | Mandatory | Food | Education/ training, labelling | Not reported | Not reported | Effective |
| Buonocore 2017 | United Kingdom | Quantitative | Cross-sectional, observational | Outcome/ impact | Compliance, environment | Chemical (Hazard Information and Packaging for Supply) Regulations 2009 (CHIP) and Classification, Labelling and Packaging Regulations (CLP) [CLP replaced CHIP in June 2015] | Regulation | Government | Mandatory | Tobacco | Education/ training, labelling | Not reported | Present | Partial effect |
| Campbell 2013 | United States | Mixed | Interviews, survey | Process | Implementation, acceptance | Philadelphia Lead Court & Philadelphia Health Code | Specialist court; Code | Government | Mandatory | Environmental pollutant | Availability, reporting | Not reported | Present | Effective |
| Chriqui 2014 | United States | Qualitative | Document analysis | Formative | Implementation, reach | USDA Snack Food and Beverage Standards | Standard | Government | Mandatory | Food | Availability | Not reported | Not reported | Effective |
| Cole 2011 | Canada | Mixed | Before and after | Process & outcome/ impact | Acceptance, compliance, economic, environment | Bylaw No. 456-2003 | Bylaw | Government | Mandatory | Environmental pollutant | Availability, education/ training | Comprehensive | Present | Effective |
| Condon-Paoloni 2015 | Australia | Mixed | Cross-sectional, observational, interviews | Process | Acceptance, implementation | Standard 1.2.7: Nutrition, health and related claims (Australia New Zealand Food Standards Code) | Standard | Government | Mandatory | Food | Marketing, labelling | Comprehensive | Present | Not effective |
| Corvalan 2019 | Chile | Qualitative | Case study (regulatory analysis) | Process | Implementation, acceptance | Law 20 606 & Decree 13/15 | Law & Decree | Government | Mandatory | Food | Availability, marketing, labelling | Comprehensive | Present | Not effective |
| Cox 2010 | United States | Quantitative | Cross-sectional, observational | Outcome/ impact | Compliance | Health and Safety Code sections 25214.1-25214.4.2 | Code | Government | Mandatory | Environmental pollutant | Availability | Comprehensive | Present | Effective |
| DeVocht 2016 | United Kingdom | Quantitative | Observational, longitudinal | Process & outcome/ impact | Implementation, health | Guidance issued under Section 182 of the Licensing Act2003 | Act & Guideline | Government | Mixed | Alcohol | Availability | Not reported | Absent | Effective |
| DiFranza 2005 | United States | Mixed | Document analysis | Process | Implementation | Tobacco Regulation for Substance Abuse Prevention and Treatment Block Grants | Grant funding | Government | Mandatory | Tobacco | Availability | Comprehensive | Present | Effective |
| DiFranza 2009 | United States | Quantitative | Before and after | Outcome/ impact | Behaviour | Alcohol, Drug Abuse, and Mental Health Administration Reorganization Act (106 STAT. 394. Synar Amendment) | Act | Government | Mandatory | Tobacco | Availability | Comprehensive | Present | Effective |
| Donovan 2007 | Australia | Mixed | Content analysis | Outcome/ impact | Compliance, environment | Alcoholic Beverages Advertising Code (ABAC). | Code | Self-regulation | Voluntary | Alcohol | Marketing | Partial | Absent | Not effective |
| Eby 2013 | United States | Mixed | cross-sectional | Process | Implementation | Tobacco free services Title 14 NYCRR Part 856 (Office of Alcoholism and Substance Abuse Services Tobacco Free Regulation) | Regulation | Government | Mandatory | Tobacco | Availability | Not reported | Not reported | Effective |
| Faber 2019 | United Kingdom | Quantitative | Cross-sectional | Outcome/ impact | Health | The Smoke-free (Private Vehicles) Regulations 2015 | Regulation | Government | Mandatory | Tobacco | Availability | Not reported | Present | Not effective |
| Fielder 2009 | Australia | Quantitative | Cross-sectional, content analysis | Outcome/ impact | Environment, compliance | The Alcohol Beverage Advertising Code (ABAC) & AANA Code of Ethics | Code | Self-regulation | Voluntary | Alcohol | Marketing | Partial | Present | Not effective |
|  |  |  |  |  | environment | Television Industry Code of Practice, Children's Television Standards | Code & standard | Co-regulation | Mandatory | Alcohol | Marketing | Not reported | Present | Not effective |
| Frieden 2005 | United States | Quantitative | Before and after | Outcome/ impact | Behaviour, socio-economic considerations | New York City Tobacco Control Strategy | Strategy | Government | Mixed | Tobacco | Availability, price, marketing, education/ training | Partial | Not reported | Effective |
| Fry 2017 | Australia | Quantitative | Cross-sectional | Outcome/ impact | compliance, environment, socio-economic considerations | Tobacco Retailer Notification (TRN) scheme | Scheme | Government | Mandatory | Tobacco | Availability | Not reported | Absent | Effective |
| Gonzalez 2013 | Netherlands | Mixed | Document analysis, interviews | Process | Implementation, acceptance | The Tobacco Act | Act | Government | Mandatory | Tobacco | Availability | Partial | Present | Partial effect |
| Gorski 2016 | United States | Quantitative | Time series analysis | Outcome/ impact | Compliance, environment | 105 CMR 225.000 Nutrition standards for competitive foods and beverages in public schools (administered under state law MGL c. 111,x223) | Act | Government | Mandatory | Food | Availability | Absent | Absent | Effective |
| Gosselt 2012 | Netherlands | Mixed | Interviews, observational | Process & outcome/ impact | Implementation, acceptance, compliance | Licensing and Catering Act | Act | Government | Mandatory | Alcohol | Availability, education/ training | Partial | Present | Not effective |
| Gruner 2018 | United States | Quantitative | Cross-sectional, observational | Formative | reach, socio-economic considerations | Nutrition Labeling of Standard Menu Items in Restaurants and Similar Retail Food Establishments (final rule) | Rule | Government | Mandatory | Food | Labelling | Not reported | Not reported | Not effective |
| Hadfield 2015 | United Kingdom | Mixed | Interviews, document analysis, cross-sectional | Process & outcome/ impact | Implementation, compliance | The Licensing Act 2003 | Act | Government | Mandatory | Alcohol | Availability, education/ training | Absent | Present | Not effective |
|  |  |  |  |  | Implementation, compliance | Social Responsibility Standards for the Production and Supply of Alcohol Drinks (SRS) | Standard | Self-regulation | Voluntary | Alcohol | Availability, education/ training | Absent | Absent | Not effective |
| Hanh 2011 | United States | Quantitative | Time series analysis | Outcome/ impact | Health | Clean Indoor Air Ordinance, Ch. 14, §§97-104 (July 1, 2003) | Ordinance | Government | Mandatory | Tobacco | Availability | Not reported | Not reported | Partial effect |
| Hammer 2018 | United States | Mixed | Case study (regulatory analysis) | Process | Implementation | Noise Control Act 1972 | Act | Government | Mandatory | Environmental pollutant | Availability | Not reported | Not reported | Not effective |
|  | Netherlands |  |  |  | Implementation | Dutch States General Noise Abatement Act of 1976 | Act | Government | Mandatory | Environmental pollutant | Availability | Comprehensive | Not reported | Effective |
|  | United Kingdom |  |  |  | Implementation | Noise Abatement Act/ Noise Policy Statement for England | Act & policy statement | Government | Mandatory | Environmental pollutant | Availability | Comprehensive | Present | Partial effect |
| Harris 2013 | United States | Quantitative | Cross-sectional, observational | Process & outcome/ impact | Implementation, reach, environment | Children's Food and Beverage Advertising Initiative (CFBAI) | Initiative | Self-regulation | voluntary | Food | Marketing | Not reported | Absent | Not effective |
| Hippensteel 2019 | United States | Quantitative | Cross-sectional, observational | Formative | Reach, socio-economic considerations | Baltimore City Zoning Code (Ordinance 16-581 - TransForm Baltimore) | Code | Government | Mandatory | Alcohol | Availability | Not reported | Not reported | Effective |
| Jensen 2015 | European Union | Mixed | Case study (regulatory analysis) | Process | Implementation, reach | The EU Pledge for responsible food marketing | Pledge | Self-regulation | Voluntary | Food | Marketing | Partial | Not reported | Not effective |
| Jiménez Ruiz 2014 | Spain | Quantitative | Cross-sectional | Outcome/ impact | Behaviour, environment | Law 28/2005 | Law | Government | mandatory | Tobacco | Availability | Not reported | Not reported | Effective |
|  |  |  |  |  | Behaviour, environment | Law 42/2010 | Law | Government | mandatory | Tobacco | Availability | Not reported | Not reported | Effective |
| Jimenez-Aguilar 2017 | Mexico | Quantitative | Cross-sectional | Outcome/ impact | Compliance, environment | General Guidelines for Dispensing or Distribution of Foods and Beverages at School Food Establishments in Elementary Schools | Guideline | Government | Voluntary | Food | Availability | Absent | Absent | Not effective |
| Jones 2016 | Australia | Qualitative | Case study (regulatory analysis) | Process | Implementation, reach | Food and Health Dialogue | Dialogue | Quasi-regulation | Voluntary | Food | Education/ training, reformulation | Not reported | Absent | Not effective |
| Jones 2007 | Australia | Mixed | Cross-sectional, observational | Process & outcome/ impact | Reach, compliance, environment | New South Wales Liquor Industry Code of Practice for Responsible Promotion of Liquor Products | Code | Co-regulation | Voluntary | Alcohol | Marketing | Absent | Present | Not effective |
| Kaplan 2019 | Turkey | Quantitative | Cross-sectional | Outcome/ impact | environment, behaviour | Law No. 5727 Prevention and Control of Hazards of Tobacco Products | Law | Government | mandatory | Tobacco | Availability | Not reported | Not reported | Partial effect |
| Kim 2013 | South Korea | Mixed | Before and after | Outcome/ impact | environment, economic | Special Act on Safety Management of Children's Dietary Life 2008 | Act | Government | Mandatory | Food | Marketing | Not reported | Present | Effective |
| King 2013 | Australia | Quantitative | Time series analysis | Outcome/ impact | Environment | Responsible Children's Marketing Initiative (RCMI); Quick Service Restaurant Industry Initiative (QSRII) for Responsible Advertising and Marketing to Children | Initiative | Self-regulation | Voluntary | Food | Marketing | Partial | Not reported | Not effective |
| Laska 2019 | United States | Quantitative | Before and after | Outcome/ impact | Compliance, environment, behaviour | Minneapolis Staple Food Ordinance, 2015 | Ordinance | Government | mandatory | Food | Availability | Comprehensive | Present | Not effective |
| Lawrence 2019 | Australia & New Zealand | Qualitative | Case study (regulatory analysis) | Process | Implementation, reach | Food Standards Australia New Zealand Act 1991; Australia New Zealand Food Standards Code | Act & standard | Government | mandatory | Food | Availability | Not reported | Present | Effective |
| Lee 2015 | United States | Quantitative | cross-sectional | Outcome/ impact | compliance, socio-economic considerations | Family Smoking Prevention and Tobacco Control Act 2009 | Act | Government | mandatory | Tobacco | Marketing, availability, labelling | Comprehensive | Present | Effective |
| León-Flández 2017 | Spain | Quantitative | cross-sectional | Outcome/ impact | Compliance, environment | Publicidad, Actividad, Obesidad, Salud [PAOS] Code | Code | Self-regulation | Voluntary | Food | Marketing | Partial | Present | Not effective |
| Lessard 2013 | United States | Quantitative | Cross-sectional, observational | Process & outcome/ impact | Implementation, compliance | 933 DELACARE: Regulations for Early Care and Education and School-Age Centers | Regulation | Government | Mandatory | Food | Availability | Comprehensive | Not reported | Effective |
| Levay 2020 | Canada | Qualitative | Interviews, survey, observational | Process | Implementation, acceptance | Guidelines for Food and Beverage Sales in British Columbia Schools | Guideline | Government | mandatory | Food | Availability | Absent | Absent | Partial effect |
| Lie 2016 | Netherlands | Qualitative | Interviews, document analysis | Process | Implementation, acceptance | 2001/37/EC Tobacco ProductsDirective (TPD) | Directive | Government | Mandatory | Tobacco | Labelling | Not reported | Not reported | Partial effect |
| Lindblom 2020 | United States | Qualitative | case study (regulatory analysis) | Process | Implementation, reach | Family Smoking Prevention and Tobacco Control Act 2009 | Act | Government | mandatory | Tobacco | Availability, labelling, price | Not reported | Present | Not effective |
| Lindell 2001 | United States | Mixed | Case study (legal analysis) | Process & outcome/ impact | Implementation, acceptance, compliance | Emergency Planning and Community Right to Know Act of 1986 | Act | Government | Mandatory | Environmental pollutant | Availability, education/ training, reporting | Comprehensive | Present | Effective |
| Long 2013 | United States | Quantitative | Time series analysis | Process & outcome/ impact | Implementation, economic, reach | Healthy Food Certification | Certification | Government | Voluntary | Food | Availability | Comprehensive | Present | Effective |
| MacKay 2009 | Australia | Qualitative | Case study (legal analysis) | Process | Implementation, acceptance, reach | Australian Association of National Advertisers Food and Beverages Advertising and Marketing Communications Code | Code | Self-regulation | Voluntary | Food | Marketing | Partial | Absent | Not effective |
| Magnusson 2015 | Australia | Qualitative | case study (regulatory analysis) | Process | Implementation, reach | Food and Health Dialogue | Dialogue | Quasi-regulation | voluntary | Food | Reformulation | Partial | Absent | Not effective |
|  | United Kingdom |  |  |  | Implementation, reach | Food Standards Authority salt reduction program | Program | Quasi-regulation | voluntary | Food | Reformulation | Comprehensive | Absent | Not effective |
| Marsh 2012 | New Zealand | Quantitative | cross-sectional | Outcome/ impact | Behaviour, socio-economic considerations and compliance | Smokefree Environments and Regulated Products Act 1990 | Act | Government | Mandatory | Tobacco | Availability | Comprehensive | Not reported | Partial effect |
| Marti­nez 2009 | Spain | Quantitative | cross-sectional | Process | Implementation, reach | Law 28/2005 | Law | Government | mandatory | Tobacco | Availability | Partial | Not reported | Effective |
| Matthews 2011 | United Kingdom | Mixed | Longitudinal, before and after | Process & outcome/ impact | environment, compliance, acceptance | The Education (Nutritional Standards and Requirements for School Food) (England) Regulations 2007 | Regulation | Government | mandatory | Food | Availability | Not reported | Not reported | Effective |
| McGuire 2020 | United States | Mixed | cross-sectional | Process | Acceptance | 2014 Minneapolis Staple Foods Ordinance | Ordinance | Government | mandatory | Food | Availability | Comprehensive | Present | Effective |
| McNabola 2012 | Ireland | Mixed | Cross-sectional, observational | Outcome/ impact | Environment | Public Health (tobacco) (amendment) Act, No. 6 of 2004 | Act | Government | mandatory | Tobacco | Availability | Not reported | Not reported | Effective |
| McNamara 2017 | Ireland | Quantitative | cross-sectional | Process | Implementation, acceptance | Safety Health and Welfare at Work Act 2005 Risk Assessment Document and statutory Code of Practice | Act & code of practice | Government | Mandatory | Environmental pollutant | Availability, education/ training, reporting | Comprehensive | Present | Effective |
| Montini 2008 | United States | Qualitative | interviews | Process | Implementation | California Labor Code 6404.5 | Code | Government | Mandatory | Tobacco | Availability | Not reported | Present | Effective |
| Murphy-Greene 2002 | United States | Quantitative | Cross-sectional, observational | Process & outcome/ impact | Implementation, environment, compliance, socio-economic considerations | Florida Statute Chapter 487 Section I (the Florida Pesticide Law) and Section II (the Florida Agricultural Worker Safety Act) | Act & standard | Government | mandatory | Environmental pollutant | Education/ training | Comprehensive | Not reported | Not effective |
| Nagami 2000 | United States | Mixed | Case study (legal analysis) | Process & outcome/ impact | Implementation, acceptance, economic, compliance | California Smoke-Free Workplace Act (AB 13) | Act | Government | Mandatory | Tobacco | Availability | Partial | Present | Effective |
| O'Brien 2019 | Australia | Qualitative | Case study (legal analysis) | Process | Implementation, reach | The DrinkWise Labelling Scheme | Scheme | Self-regulation | voluntary | Alcohol | Labelling | Absent | Absent | Not effective |
| Oleinick 2014 | United States | Quantitative | Case-control | Outcome/ impact | Health | Chemical Hazard Communication Standard | Standard | Government | mandatory | Environmental pollutant | Labelling, education/ training | Comprehensive | Not reported | Effective |
| Olstad 2013 | Canada | Qualitative | Interviews | Process | Implementation, acceptance | Alberta Nutrition Guidelines for Children and Youth | Guideline | Government | voluntary | Food | Availability | Not reported | Not reported | Not effective |
| Patterson 2015 | Sweden | Quantitative | Cross-sectional | Outcome/ impact | Compliance, environment | Education Act | Act | Government | mandatory | Food | Availability | Comprehensive | Not reported | Effective |
| Peeters 2013 | European Union | Mixed | cross-sectional | Outcome/ impact | Compliance | 2001/37/EC Tobacco Products Directive (TPD) | Directive | Government | Mandatory | Tobacco | Availability | Not reported | Not reported | Not effective |
|  |  |  |  |  | Compliance | Directive 2008/118/EC | Directive | Government | Mandatory | Tobacco | Price | Not reported | Not reported | Not effective |
|  |  |  |  |  | Compliance | Directive 2003/33/EC | Directive | Government | Mandatory | Tobacco | Marketing | Not reported | Not reported | Not effective |
| Phetxumphou 2016 | United States | Mixed | Content analysis | Process | Implementation | Safe Drinking Water Act | Act | Government | mandatory | Environmental pollutant | Education/ training, reporting | Partial | Present | Not effective |
| Phillips 2010 | United States | Quantitative | cross-sectional | Process & outcome/ impact | Implementation, environment | Arkansas Legislative Act 1220 of 2003 | Act | Government | mandatory | Food | Availability, reporting | Not reported | Not reported | Effective |
| Pierce 2019 | Australia | Mixed | case study (regulatory analysis) | Process | Implementation, reach | Alcohol Beverages Advertising Code (ABAC) Scheme | Code | Self-regulation | Voluntary | Alcohol | Marketing | Partial | Absent | Not effective |
| Pierce 2017 | Australia | Qualitative | case study (regulatory analysis) | Process | Implementaion | Alcohol Advertising Review Board Code | Code | Self-regulation | voluntary | Alcohol | Marketing | Partial | Present | Effective |
| Pimentel 2019 | United States | Quantitative | Cross-sectional | Outcome/ impact | Environment | Tobacco Free Pharmacies: SF Health Article Code 19J, Sec. 1009.92 (2008) | Ordinance | Government | Mandatory | Tobacco | Availability | Not reported | Not reported | Effective |
| PotvinKent 2019 | Canada | Quantitative | cross-sectional | Process & outcome/ impact | Implementation, acceptance, reach, environment | Children's Food and Beverage Advertising Initiative | Initiative | Self-regulation | voluntary | Food | Marketing | Not reported | Not reported | Not effective |
| Probart 2010 | United States | Mixed | cross-sectional | Process & outcome/ impact | Implementation, environment | The Child Nutrition and Women, Infants, and Children Reauthorization Act 2004 | Act | Government | mandatory | Food | Availability | Partial | Not reported | Partial effect |
| Provost 2019 | United States | Quantitative | Cross-sectional, document analysis | Process | Implementation | Executive Order 12898, Federal Actions to Address Environmental Justice in Minority Populations and Low-Income Populations (1994) | Executive Order | Government | Mandatory | Environmental pollutant | Education/ training, reporting | Comprehensive | Not reported | Not effective |
| Pulker 2018 | Australia | Mixed | cross-sectional | Process & outcome/ impact | Implementation, reach, compliance, environment | Australia New Zealand Food Standards Code | Code | Government | Mandatory | Food | Labelling | Not reported | Not reported | Not effective |
|  | United States |  |  |  | Implementation, reach | Health Star Rating Scheme | Scheme | Government | Voluntary | Food | Labelling | Not reported | Not reported | Not effective |
| Rajkumar 2015 | Switzerland | Quantitative | Longitudinal, before and after | Process & outcome/ impact | Implementation, acceptance, compliance | Swiss Tobacco Control Act | Act | Government | mandatory | Tobacco | Availability | Not reported | Not reported | Effective |
| Ramos 2015 | Spain | Quantitative | Observational | Outcome/ impact | Compliance, environment | Publicidad, Actividad, Obesidad, Salud [PAOS] Code | Code | Self-regulation | voluntary | Food | Marketing | Not reported | Not reported | Not effective |
| Rappazzo 2007 | United States | Quantitative | Time series analysis | Outcome/ impact | Health | Childhood Lead Poisoning Prevention Program of Philadelphia | Program | Government | Mixed | Environmental pollutant | Availability | Comprehensive | Present | Not effective |
| Reeve 2013 | Australia | Qualitative | case study (regulatory analysis) | Process | Implementation, reach | Responsible Children’s Marketing Initiative (RCMI); Quick Service Restaurant Initiative for Responsible Advertising and Marketing to Children (QSRI) | Initiative | Self-regulation | voluntary | Food | Marketing | Partial | Present | Not effective |
| Reeve 2015 | United States | Mixed | case study (regulatory analysis) | Process | Implementation, reach | National Salt Reduction Initiative (NSRI) | Initiative | quasi-regulation | Voluntary | Food | Reformulation | Partial | Absent | Not effective |
|  | United Kingdom |  |  |  | Implementation | Food Standards Authority salt reduction program | Program | quasi-regulation | Voluntary | Food | Reformulation | Partial | Absent | Not effective |
| Reichmann 2012 | Austria | Quantitative | Cross sectional, observational | Process & outcome/ impact | Implementation, acceptance, compliance, economic | Austrian Tobacco Act | Act | Government | mandatory | Tobacco | Availability | Not reported | Not reported | Not effective |
| Ritchie 2015 | United States | Quantitative | cross-sectional | Process & outcome/ impact | Implementation, environment | The California Healthy Beverages in Childcare Law | Act | Government | mandatory | Food | Availability | Not reported | Not reported | Effective |
| Roberto 2012 | United States | Quantitative | Cross sectional, observational | Process | Implementation, reach | Smart Choices' labelling programme | Program | Self-regulation | voluntary | Food | Labelling | Not reported | Not reported | Not effective |
| Roberts 2012 | Australia | Quantitative | longitudinal | Outcome/ impact | Compliance, environment | Children’s Television Standards (CTS) | Standard | Co-regulation | Mandatory | Food | Marketing | Not reported | Not reported | Not effective |
|  |  |  |  |  | Compliance, environment | Responsible Children's Marketing Initiative (RCMI); Quick Service Restaurant Initiative for Responsible Advertising and Marketing to Children (QSRI) | Initiative | Self-regulation | Voluntary | Food | Marketing | Partial | Not reported | Not effective |
| Rodríguez 2019 | Spain | Quantitative | Cross-sectional | Outcome/ impact | Compliance, environment | Publicidad, Actividad, Obesidad, Salud [PAOS] Code | Code | Self-regulation | voluntary | Food | Marketing | Not reported | Not reported | Not effective |
| Romero-FernÃ¡ndez 2010 | Spain | Quantitative | Cross-sectional, observational | Outcome/ impact | Compliance | Publicidad, Actividad, Obesidad, Salud [PAOS] Code | Code | Self-regulation | voluntary | Food | Marketing | Partial | Present | Not effective |
| Roodbeen 2018 | Netherlands | Mixed | Interviews, observational | Process & outcome/ impact | Implementation, compliance | Dutch Licensing and Catering Act (DLCA) | Act | Government | mandatory | Alcohol | Availability | Partial | Present | Partial effect |
| Ross 2016 | United States | Quantitative | longitudinal | Outcome/ impact | Compliance, environment, economic | Distilled Spirits Council of the United States Code of Responsible Practices for Beverage Alcohol Advertising and Marketing; The Beer Institute Advertising and Marketing Code; The Wine Institute Code of Advertising Standards | Code & Guideline | Self-regulation | Voluntary | Alcohol | Marketing | Not reported | Not reported | Not effective |
| Ross 2017 | United States | Quantitative | longitudinal | Outcome/ impact | Compliance, environment | Distilled Spirits Council of the United States Code of Responsible Practices for Beverage Alcohol Advertising and Marketing; The Beer Institute Advertising and Marketing Code; The Wine Institute Code of Advertising Standards | Code | Self-regulation | voluntary | Alcohol | Marketing | Not reported | Not reported | Partial effect |
| Rychert 2020 | New Zealand | Qualitative | interviews | Process | Implementation, acceptance | Alcohol licensing trusts - under the Sale and Supply of Alcohol Act 2012 | Act & trust | Co-regulation | Mixed | Alcohol | Availability | Comprehensive | Not reported | Partial effect |
| Sainsbury 2017 | Australia | Quantitative | Cross-sectional, observational | Process & outcome/ impact | reach, environment | Australian Association of National Advertisers Code of Ethics; Australian Association of National Advertisers Food & Beverages Advertising & Marketing Communications Code; Australian Association of National Advertisers Code of Advertising & Marketing Communications to Children; Responsible Children’s Marketing Initiative (RCMI); Quick Service Restaurant Initiative for Responsible Advertising and Marketing to Children (QSRI) | Code | Self-regulation | voluntary | Food | Marketing | Not reported | Not reported | Not effective |
| Sanchez-Vaznaugh 2015 | United States | Quantitative | cross-sectional | Outcome/ impact | Health, socio-economic considerations | The California Childhood Obesity Prevention Act (SB 677, 2003) | Act | Government | mandatory | Food | Availability | Not reported | Not reported | Effective |
| Sato 2018 | Japan | Quantitative | Cross-sectional, observational | Process | Implementation | The Hyogo Prefectural Ordinance on the Prevention of Exposure to Second-hand Smoke 2012 | Ordinance | Government | mandatory | Tobacco | Availability | Not reported | Present | Not effective |
| Scott 2008 | United States | Quantitative | Cross-sectional, observational | Process & outcome/ impact | Implementation, compliance, environment, socio-economic considerations | Outdoor Advertising Association of America Code of Industry Principles | Code | Self-regulation | voluntary | Alcohol & Tobacco | Marketing | Absent | Absent | Not effective |
| Shipley 2008 | United Kingdom | Mixed | Cross-sectional, observational | Process | Implementation, acceptance | Smoke-free (Premises and Enforcement) Regulations 2006 | Regulation | Government | mandatory | Tobacco | Availability | Not reported | Not reported | Not effective |
| Sing 2020 | New Zealand | Qualitative | case study (regulatory analysis) | Process | Implementation, reach | Children and Young People's Advertising Code (CYPA Code) | Code | Self-regulation | voluntary | Food | Marketing | Partial | Present | Not effective |
| Smith 2014 | United States | Quantitative | Observational | Process & outcome/ impact | Implementation, compliance, environment | Alcohol and Tobacco Tax and Trade Bureau Regulations (27 CFR chapter 1) | Regulation | Government | Mandatory | Alcohol | Marketing | Not reported | Not reported | Partial effect |
|  |  |  |  |  | Implementation, compliance, environment | Distilled Spirits Council of the United States Code of Responsible Practices for Beverage Alcohol Advertising and Marketing; The Beer Institute Advertising and Marketing Code; | Code | Self-regulation | Voluntary | Alcohol | Marketing | Not reported | Absent | Not effective |
| Spivak 2015 | United States | Quantitative | Linear modelling | Outcome/ impact | Compliance, behaviour, socio-economic considerations | Alcohol, Drug Abuse, and Mental Health Administration Reorganization Act (106 STAT. 394. Synar Amendment) | Act | Government | mandatory | Tobacco | Availability | Comprehensive | Present | Effective |
| Stoltze 2019 | Chile | Quantitative | Before and after, observational | Process & outcome/ impact | Implementation, environment | Food Labeling and Advertising Regulation (Law 20.606) | Regulation | Government | mandatory | Food | Availability, marketing | Not reported | Not reported | Effective |
| Strauss 2011 | United States | Qualitative | Case-study (legal analysis) | Formative | Implementation, reach | Food Safety Modernization Act | Act | Government | Mandatory | Food | Availability | Comprehensive | Present | Effective |
| Sureda 2014 | Spain | Quantitative | cross-sectional | Outcome/ impact | Behaviour, environment | Law 42/2010 | Law | Government | mandatory | Tobacco | Availability | Comprehensive | Present | Effective |
| Szanyi 2010 | United States | Mixed | Case-study (legal analysis) | Process | Implementation, reach | New York City Health Code (menu labelling provision) | Code | Government | Mandatory | Food | Labelling | Not reported | Not reported | Effective |
|  | United Kingdom |  |  |  | Implementation, reach | Front of pack food labelling | Scheme | Government | Voluntary | Food | Labelling | Not reported | Not reported | Partial effect |
| Thomson 2005 | New Zealand | Qualitative | Document analysis, interviews | Process | Implementation, reach | Fair Trading Act 1986 | Act | Government | Mandatory | Tobacco | Availability, education/training, reporting | Not reported | Absent | Not effective |
|  |  |  |  |  | Implementation, reach | Smoke-free Environments Act 1990 | Act | Government | Mandatory | Tobacco | Availability, education/training, reporting | Not reported | Absent | Not effective |
| VanderWekken 2012 | Canada | Qualitative | interviews | Process | Implemetation, acceptance | Healthier Choices in  Vending Machines in BC  Public Buildings Policy | Guideline | Government | voluntary | Food | Availability | Not reported | Not reported | Effective |
| Vilaro 2017 | United States | Quantitative | Observational | Process & outcome/ impact | Implementation, environment | Children's Food and Beverage Advertising Initiative (CFBAI) | Initiative | Self-regulation | voluntary | Food | Marketing | Partial | Present | Not effective |
| Wellard-Cole 2019 | Australia | Qualitative | Observational, content analysis | Process | Implementation | Standard 1.2.7: Nutrition, health and related claims (Australia New Zealand Food Standards Code) | Standard | Government | mandatory | Food | Marketing | Partial | Present | Partial effect |
| Wilkinson 2020 | Australia | Qualitative | interviews | Process | Implementation, acceptance, reach | Practice Note 61 Licensed Premises: Assessing cumulative impact | Practice Note | Government | Mixed | Alcohol | Availability | Not reported | Not reported | Not effective |
| Williams 2004 | United States | Quantitative | Cross-sectional, observational | Process & outcome/ impact | Implementation, acceptance, compliance | New Hampshire Indoor Smoking Act | Act | Government | Mandatory | Tobacco | Availability | Partial | Present | Not effective |
| Yorifuji 2011 | Japan | Quantitative | Cross-sectional, observational | Outcome/ impact | Compliance, health, behaviour | 2003 Health Promotion Law (Article 25) | Act | Government | mandatory | Tobacco | Availability | Not reported | Present | Effective |
| Zacher 2013 | Australia | Quantitative | before and after | Outcome/ impact | Compliance | Tobacco Amendment (Protection of Children) Act 2009 | Act | Government | mandatory | Tobacco | Marketing, price | Comprehensive | Not reported | Effective |
