# Supplementary material for: Legal and regulatory instruments for NCD prevention: a scoping review and descriptive analysis of evaluations in OECD countries
Source: BMC Public Health. 2024 Feb 29;24:641. doi: 10.1186/s12889-024-18053-4 (PMC10903077; doi:10.1186/s12889-024-18053-4)
Supplement: Supplementary file 6 — Additional file 6 [file 12889_2024_18053_MOESM6_ESM.docx]

Additional file 1: Search strategy - key concepts and keywords/synonyms used across all databases

| Evaluation | Public health  risks | Instrument | Governance |
| --- | --- | --- | --- |
| evaluat*  analys*  analyz*  outcome*  effect*  review  impact  design  implement* | “public health”  Food  Sugar  Salt  Drink  Beverage  Nutri*  Alcohol*  Liquor  Tobacco  Cigarette*  “environmental hazard”  Lead  Asbestos  chemical | legislat*; law*  regulat*; compliance  “code* of practice”  standard*  guideline*  scheme  “legal tool”  “legal instrument”  health policy/health policies | government*  government regulation*  governance  policy; policies; policymaker*  co-regulat*  co regulat*  self-regulat*  self regulat*  private governance  independent* |
| Scopus | | | |
| ((TITLE-ABS-KEY("legislat*" OR "law" OR "regulat*" OR "compliance*" OR "code* of practice" OR “standard*” OR “guideline*” OR “scheme” OR “legal tool” OR “legal instrument” OR “health polic*”)) AND ((TITLE-ABS-KEY("government*" OR “governance” OR “policy*” OR “policies” OR “co-regulat*” OR “co regulat*” OR “self-regulat* OR “self regulat*” OR “private govern*” OR “independent*”)) AND ((TITLE-ABS-KEY("food" OR “sugar” OR “salt” OR “drink” OR “beverage” OR “nutri* OR “alcohol*” OR “liquor” OR “cigarette* OR “smoke” OR “environmental hazard” OR “lead” OR “chemical” OR “asbestos”)) AND ((TITLE-ABS-KEY("evaluat*” OR “analy*” OR “outcome*” OR “effect*” OR “review" OR “impact” OR “design” OR “implement*”)) AND ((TITLE-ABS-KEY("public health")) AND (PUBYEAR > 2000) AND (LIMIT-TO(LANGUAGE, "English")) AND (LIMIT-TO (EXACTKEYWORD, "Public health")) | | | |
| HeinOnline | | | |
| (((((((((((("legislat*") OR ("law")) OR ("regulat*")) OR ("compliance*")) OR ("code* of practice")) OR (“standard*”)) OR (“guideline*”)) OR (“scheme”)) OR (“legal tool”)) OR (“legal instrument”)) OR (“health polic*”))) AND (((((((((("government*") OR (“governance”)) OR (“policy*”)) OR (“policies”)) OR (“co-regulat*”)) OR (“co regulat*”)) OR (“self-regulat*)) OR (“self regulat*”)) OR (“private govern*”)) OR (“independent*”))) AND ((((((((((((((("food") OR (“sugar”)) OR (“salt”)) OR (“drink”)) OR (“beverage”)) OR (“nutri*)) OR (“alcohol*”)) OR (“liquor”)) OR (“cigarette*)) OR (“smoke”)) OR (“environmental hazard”)) OR (“lead”)) OR (“chemical”)) OR (“asbestos”))) AND ((((((((("evaluat*”)) OR (“analy*”)) OR (“outcome*”)) OR (“effect*”)) OR (“review")) OR (“impact”)) OR (“design”)) OR (“implement*”))) AND (("public health")) in All Databases.  Limit to English  Limit to published >2000 | | | |
| EMBASE | | | |
| 1. Public health.mo or public health/ 2. Law.mp or Jurisprudence/ 3. Legal instrument.mp or Health Policy/ 4. (standard or “code* of practice” or guide* or scheme or instrument).af 5. Legislation.mp 6. Regulation.mp 7. Compliance.mp or compliance/ or legal aspect/ or enforce*.mp or law/ 8. 2 or 3 or 4 or 5 or 6 or 7 9. Self-regulat*.mp or self-control/ 10. Co-regulat*.mp 11. Governance.mp 12. Local Government/ or Federal Government/ or Government Agencies/ or State Government/ or government.mp or Government/ or Government regulation/ 13. 9 or 10 or 11 or 12 14. Evaluat*.mp or evaluation/ 15. Effect*.mp 16. Impact.mp or impact assessment/ 17. Risk benefit analysis/ or analysis or analysis.mp 18. Review.mp or “institutional review”/ or “review”/ or “utilization review”/ 19. Design.mp or design/ 20. Implement*.mp 21. 14 or 15 or 16 or 17 or 18 or 19 or 20 22. Food quality/ or food industry/ or food quantity/ or food.mp or food poisoning/ or hospital food service/ or baby food/ or processed food/ or food processing/ or ultra-processed food/ or junk food/ or food storage/ or food packaging/ or food preservation/ or “takeaway (food)”/ or minimally processed food/ or food contamination/ or food crop/ or food organism/ or food processing waste/ or food security/ or “Food and Drug Administration”/ or food ingredient/ or preserved food/ or food/ or food guide pyramid/ or food consumption/ chain/ or food insecurity/ or food composition/ or food availability/ or food control/ or convenience food/ or food standard/ or fast food/ 23. Alcohol consumption/ or alcohol.mp or alcohol/ or alcohol production/ or alcohol intoxication/ or alcohol abuse/ 24. “tobacco use”/ or tobacco/ or tobacco industry/ or tobacco smoke/ or tobacco.mp or tobacco consumption/ 25. Environmental hazard.mp or occupational exposure/ or occupational health/ or injury/ 26. Lead poisoning/ or paint.mp or paint industry/ or paint/ or air pollution/ or chemical/ 27. Asbestos.mp or asbestos/ or asbestos fiber/ 28. 22 or 23 or 24 or 25 or 26 or 27 29. 8 and 13 and 21 and 28 30. Limit 29 to (English language and full text) | | | |
| MEDLINE | | | |
| 1. Public health.mo or public health/ 2. Law.mp or Jurisprudence/ 3. Legal instrument.mp or Health Policy/ 4. (standard or “code* of practice” or guide* or scheme or instrument).af 5. Legislation.mp or legislation as topic/ 6. Regulation.mp 7. Compliance.mp or compliance/ or legal aspect/ or enforce*.mp or law/ 8. 2 or 3 or 4 or 5 or 6 or 7 9. Self-regulat*.mp or self-control/ 10. Co-regulat*.mp 11. Governance.mp 12. Local Government/ or Federal Government/ or Government Agencies/ or State Government/ or government.mp or Government/ or Government regulation/ 13. 9 or 10 or 11 or 12 14. Evaluat*.mp or evaluation/ 15. Effect*.mp 16. Impact.mp or impact assessment/ 17. Risk benefit analysis/ or analysis or analysis.mp 18. Review.mp or “institutional review”/ or “review”/ or “utilization review”/ 19. Design.mp or design/ 20. Implement*.mp 21. 14 or 15 or 16 or 17 or 18 or 19 or 20 22. Food/ or legislation as Topic/ or food industry/ 23. Alcohol as Topic/ 24. Tobacco as Topic/ 25. Environmental pollutant at Topic/ 26. Lead poisoning/ or paint.mp or paint industry/ or paint/ or air pollution/ or chemical/ 27. Asbestos.mp or asbestos/ or asbestos fiber/ 28. 22 or 23 or 24 or 25 or 26 or 27 29. 8 and 13 and 21 and 28 | | | |
