# Supplementary material for: Legal and regulatory instruments for NCD prevention: a scoping review and descriptive analysis of evaluations in OECD countries
Source: BMC Public Health. 2024 Feb 29;24:641. doi: 10.1186/s12889-024-18053-4 (PMC10903077; doi:10.1186/s12889-024-18053-4)
Supplement: Supplementary file 7 — Additional file 7 [file 12889_2024_18053_MOESM7_ESM.docx]

*Additional file 2: Data extraction template*

| General information | Title |
| --- | --- |
|  | Date |
|  | Country |
| Characteristics of included studies | Aim of study |
|  | Evaluation type   - Process - Outcome/ impact - Economic - Formative |
|  | Study design   - Quantitative - Qualitative - Mixed methods |
|  | Methods   - Cross-sectional - Before and after - Case study - Interviews - Other |
|  | Study funding |
|  | Declared conflicts of interest |
| Legal instrument | Title |
|  | Public health risk   - Tobacco - Alcohol - Food and beverages - Environmental pollutant |
|  | Objective |
| Legal framework | Type   - Act - Regulation - Standard - Guideline - Other |
|  | Regulatory model   - government - co-regulation - quasi-regulation - self-regulation |
|  | Jurisdiction (e.g. national, state) |
| Design | Substantive features (key details of the policy e.g. target, setting, exclusions, definitions) |
|  | Policy type (e.g. availability, affordability, marketing, training, awareness raising) |
| Implementation | Voluntary or mandatory |
|  | Monitoring |
|  | Enforcement |
| Evaluation/ results | Evaluation measure   - health (e.g. disease prevalence, mortality) - behaviour (e.g. purchasing, consumption, beliefs) - environment (e.g. exposure, reformulation, education) - compliance (e.g. rates of compliance, complaints) - socio-economic considerations (e.g. reports outcomes for different populations) - economic (e.g. revenue, market share) - implementation (e.g. as intended, barriers/ enablers, independency and transparency) - acceptance (e.g. support from public, industry, public service) - reach (e.g. coverage, comprehensiveness) |
|  | Summary of key findings |
|  | Reported effectiveness |
|  | Reported criticisms |
